# Validation of quantitative fatty acid signature analysis for estimating the diet composition of free-ranging killer whales

**DOI:** 10.1038/s41598-022-11660-4

**Published:** 2022-05-13

**Authors:** Anaïs Remili, Rune Dietz, Christian Sonne, Sara J. Iverson, Denis Roy, Aqqalu Rosing-Asvid, Haley Land-Miller, Adam F. Pedersen, Melissa A. McKinney

**Affiliations:** 1 Department of Natural Resource Sciences, McGill University, Sainte-Anne-de-Bellevue, QC H9X 3V9 Canada; 2 Department of Ecoscience, Arctic Research Centre, Aarhus University, 4000 Roskilde, Denmark; 3 Department of Biology, Dalhousie University, Halifax, NS Canada; 4 Greenland Institute of Natural Resources, Nuuk, 3900 Greenland

**Keywords:** Ecological modelling, Fatty acids

## Abstract

Accurate diet estimates are necessary to assess trophic interactions and food web dynamics in ecosystems, particularly for apex predators like cetaceans, which can regulate entire food webs. Quantitative fatty acid analysis (QFASA) has been used to estimate the diets of marine predators in the last decade but has yet to be implemented on free-ranging cetaceans, from which typically only biopsy samples containing outer blubber are available, due to a lack of empirically determined calibration coefficients (CCs) that account for fatty acid (FA) metabolism. Here, we develop and validate QFASA for killer whales using full blubber from managed-care and free-ranging individuals. First, we compute full, inner, and outer blubber CCs from the FA signatures across the blubber layers of managed-care killer whales and their long-term diet items. We then run cross-validating simulations on the managed-care individuals to evaluate the accuracy of diet estimates by comparing full-depth and depth-specific estimates to true diets. Finally, we apply these approaches to subsistence-harvested killer whales from Greenland to test the utility of the method for free-ranging killer whales, particularly for the outer blubber. Accurate diet estimates for the managed-care killer whales were only achieved using killer whale-specific and blubber-layer-specific CCs. Modeled diets for the Greenlandic killer whales largely consisted of seals (75.9 ± 4.7%) and/or fish (20.4 ± 2.4%), mainly mackerel, which was consistent with stomach content data and limited literature on this population. Given the remote habitats and below surface feeding of most cetaceans, this newly developed cetacean-specific QFASA method, which can be applied to outer-layer biopsies, offers promise to provide a significant new understanding of diet dynamics of free-ranging odontocetes and perhaps other cetacean species throughout the world’s oceans.

## Introduction

Accurate diet estimates are necessary to assess trophic interactions and food web dynamics of ecosystems, particularly in the case of apex predators, which can regulate entire food webs through trophic cascades^1^. Cetaceans, especially odontocetes (toothed-whales), are at the top of the oceans’ food webs and their effects on ecosystems have been documented for decades^2^. Nonetheless, their diets and related inter- and intra-population variation are not often well known, especially in remote regions where visual observation can be challenging^3^. While visual observations of cetaceans foraging in the wild can provide valuable information on feeding ecology, data acquisition through observation of predation events is infrequent, limited to surface-events, often seasonal, and may not accurately reflect the long-term diet of a population^4^. Similarly, stomach contents and fecal samples are both challenging to obtain, only represent recent feeding patterns and, in the case of stomach contents, can only be obtained from deceased individuals that may not represent the healthy part of the populations^4^. Thus, the use of chemical tracers measured largely from biopsies collected remotely from cetaceans has increased in recent decades due to relative ease of sampling and ability to reflect integrated diet signals over time^5,6^. Stable isotopes of carbon and nitrogen in the skin have revealed some inter and intra-population variation in the feeding patterns of cetaceans, providing diet composition estimates mostly at the trophic level, although species-level precision from stable isotopes remains challenging^7–9^. And while higher-resolution fatty acid (FA) signature analysis of blubber has also been applied to a couple of cetacean populations to infer dietary patterns, quantitative estimates of prey species in their diet using FA-based approaches from biopsies of free-ranging individuals have yet to be achieved^10–13^.

FA signatures can be analyzed quantitatively to estimate the diets of predator populations, based on the knowledge that certain FAs are integrated with minor and predictable modification from the prey to the predator’s fat storage tissues (e.g., blubber). The quantitative FA signature analysis (QFASA) model was developed to estimate the combination of prey FA signatures that comes closest to matching that observed in the predator, after accounting for predator FA metabolism^14^. This model requires representative FA signatures of all major potential prey species, FA signatures of the predator, selection of an appropriate subset of diet-derived FAs to include from the total FAs monitored, species-specific calibration coefficients (CCs) to account for the predator FA metabolism, and a statistical model that minimizes the distance between the predator and the mixture of prey species representing the diet^14^. The QFASA method has been applied to a number of marine mammals including grey seals (*Halichoerus grypus*), harbour seals (*Phoca vitulina*), and polar bears (*Ursus maritimus*), providing proportional estimates of the prey species composition of these predators’ diets^14–18^. This type of analysis has not yet been applied to cetacean biopsies because CCs have not yet been determined for any cetacean species, due to the challenging aspects of managed-care (captive) feeding trials and the issue of FA stratification across cetacean blubber^10,19^.

To apply QFASA to cetaceans, cetacean-specific (or even species-specific) CCs are likely required because CCs allow the model to account for differences in the proportion of a given FA between the prey and predator due to predator-specific metabolism^14,20^. CCs are computed for each FA as the ratio of the FA proportion in the predator to the FA proportion in the prey and are required for accurate dietary estimates^14^. Feeding trials have been used in previous studies to successfully compute CCs for pinnipeds and mustelids^14,18^. While feeding trials are logistically and financially difficult to implement for cetaceans, CCs could be computed from managed-care individuals fed a constant diet over an extensive period to ensure proper and complete integration of the prey FA into the blubber.

Another challenge with applying QFASA to cetaceans, and particularly odontocetes, comes from the stratification of FAs throughout blubber layers^10,21^. Dietary FAs are more represented within the inner layer (closer to capillary and muscle layers) of the blubber and thus inner blubber has been the preferred sample for studying feeding patterns using FAs^22^. CCs are likely to vary between layers and thus blubber-layer specific CCs are likely required for cetaceans to avoid biased diet estimates. A recent study used CCs from mink (*Neovison vison*) to compare the known diets of two captive beluga whales (*Delphinapterus leucas*)^19^. Although results were promising for estimating the diets of wild belugas^23^, the potential for more accurate diet estimates using CCs developed specifically for cetaceans remains unmet. In addition, these previous beluga studies focussed only on inner blubber tissues (collected from subsistence-harvest), whereas most free-ranging cetaceans are remote biopsy darted, which only collects outer blubber (and skin). Thus, effectively applying QFASA to wild cetaceans requires investigating the predator FA signatures and determining CCs across blubber layers. Additionally, the inner and outer layers could be ecologically relevant as they may represent different feeding windows since ingested FAs are deposited more rapidly in the inner blubber^24^. A recent study reported that the inner blubber of belugas represents the diet two-to-five weeks prior to sampling^19^. If deposition follows a pattern from inner to outer layers, FA signatures in the outer layers could represent a diet integrated over a longer period, and potentially over multiple seasons^14,21^.

As the oceans’ top cetacean predator capable of highlighting strong individual feeding specializations, killer whales (*Orcinus orca*) are a prime candidate for the development of a QFASA method for cetaceans. They also have a thick layer of blubber, thus facilitating layer-specific analyses. Additionally, while feeding trials are difficult to implement, many managed-care killer whales are kept in facilities around the world, allowing access to both archived full-depth blubber samples and diet items. In this paper, we develop and validate QFASA for killer whales. First, we compute both full-depth and depth-specific CCs to account for FA metabolism in killer whales using full blubber depth FA signatures from managed-care killer whales and FA signatures from four prey species representative of their known long-term (multiple years) diets. We then run cross-validating simulations on these managed-care animals to evaluate the accuracy of the estimates by comparing the full-depth and depth-specific estimates to their known long-term diets. Finally, we apply full, inner, and outer depth approaches to samples of harvested free-ranging killer whales, to further test the applicability of the method, not just for animals with full or inner blubber available, but also from animals for which only outer blubber is collected, thus maximizing the utility of this method for feeding ecology studies on free-ranging odontocetes and possibly other cetaceans.

## Results

### Calibration coefficients

**Figure 1:**
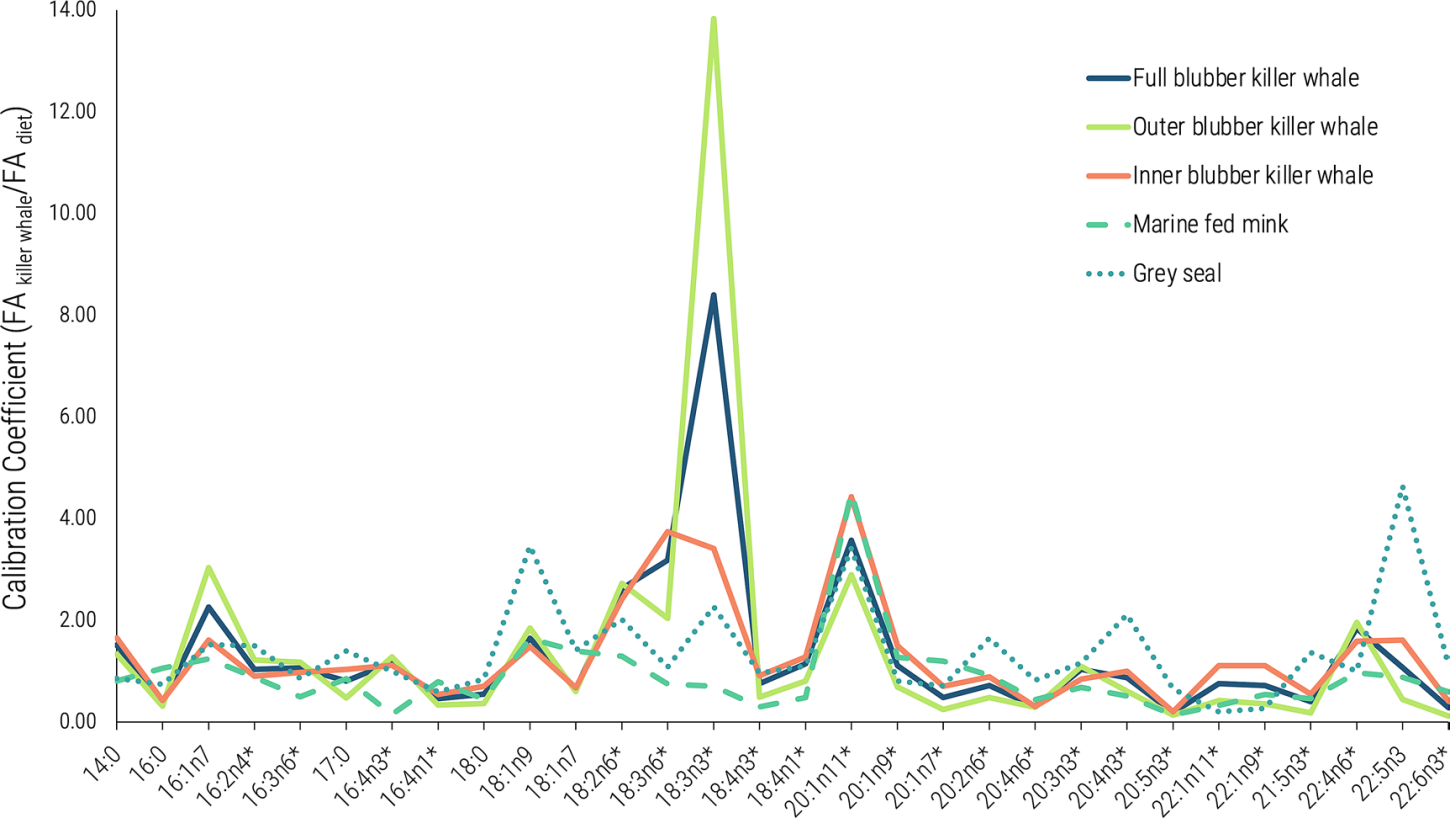
Calibration coefficients (CCs; ratio of each FA in the predator to that in its diet) used in the QFASA simulations for cetaceans. Killer whale CCs were calculated from four managed-care killer whales and their known diet species in the current study, while the grey seal CCs and the marine-fed mink CCs were reported previously (Iverson et al. 2004; Thiemann et al. 2008). CCs shown here include all dietary (with asterisk) and extended dietary FAs that were greater than 0.1% of total FAs. Dietary FAs only arise from the diet, while extended dietary FAs also include FAs partially biosynthesized by the predator.

CCs were generated from the FA signatures of the managed-care killer whales and from the FA signatures of their known diet items for each layer of the killer whales' blubber, with layers 1–4 (closer to the muscle) representing the inner blubber and layers 6–10 (closer to the skin) representing the outer blubber (Table S2). These killer whale CCs showed differences between the inner and outer blubber of the managed-care individuals, generally with the greatest differences from 1:1 for outer layers (Fig. 1, Fig. S2). The CCs calculated for the full and outer blubber of the managed-care killer whales were also distinct from those previously generated from captive feeding trials on mink and grey seal CCs, especially for 18:3n3 (Fig. 1, Table S3). Some of the inner layer killer whale CCs, however, appeared to be more similar to the CCs from mink and grey seals.

### QFASA on the managed-care killer whales

We first selected a set of FA to use for our modeling approach based on the QFASA models diagnostics (See Methods). The goodness-of-fit check for the model for the full depth blubber using the function *pred_beyond_prey* showed that 39.3% of the predator FA were outside the range of prey FA without CCs. In contrast, with CCs derived from the managed-care killer whales on the dietary FA set, only 11.9% of the predator FAs were outside the range of prey FAs, indicating an appropriate set of CCs and prey library. The other assumption of QFASA (*i.e.*: quality of the prey library) was tested using the *leave_one_prey_out* (LOPO) function on the dietary FA sets and resulted in between 84.4% and 91.2% of correct species attribution on average (KL and Aitchison distance, respectively). The extended dietary FA set (n = 30) scored similarly in the LOPO analysis, between 87.2% and 91.8% on average (Kullback–Leibler (KL) and Aitchison distance, respectively) (Table S4). The extended dietary FA set scored higher in the *pred_beyond_prey* (17.0% of predator FA outside the prey FA range), which indicates a poorer fit. Thus, although relatively similar, we selected the dietary FA set (n = 21) for subsequent analyses.

**Figure 2:**
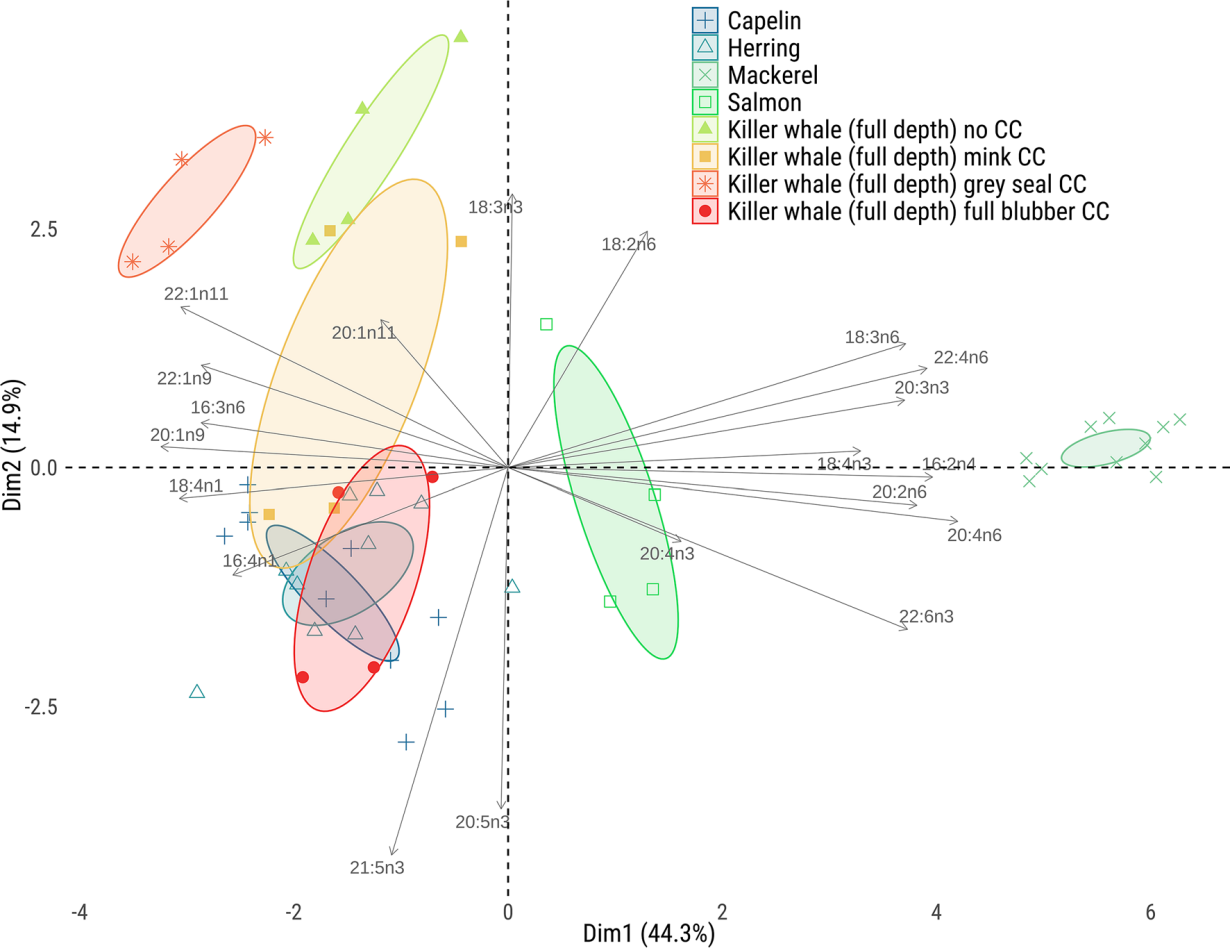
Principal component analysis (PCA) of dietary FA signatures in prey species, as well as in managed-care killer whales without CCs, with previously published mink and grey seal CCs, and with killer whale CCs generated for this study. Applying killer whale CCs to the predator moved their FA signatures within the ranges of the prey FA signatures (*i.e*.: capelin and herring mainly).

Killer whale CCs were necessary to estimate the managed-care killer whales’ diet accurately. The need for these CCs was visually supported by a PCA run on the dietary FA set for the managed-care killer whales and their prey, using various CCs (including no CCs) applied to the full blubber FA signatures (Fig. 2). Indeed, figure 2 demonstrates that applying full depth CCs to the full depth FA signatures put the predator FA signatures into the prey FA range, while no CCs or CCs from seals or mink left the full depth FA signatures well outside the prey range. Moreover, QFASA simulations on the four managed-care killer whales using no CCs resulted in a large overestimation of herring in their diets (Table S5) as herring reached a mean of 100% in the modeled diet for each killer whale, compared to 60% in their true diet. CCs calculated for non-cetacean species were also not successful at estimating the diets of the managed-care killer whales. The mink and grey seal-derived CCs showed, on average, 80.7% error compared to the true diet when the full blubber FA signatures were used, and 102.8% error using outer blubber FA signatures. Mink and grey seal CCs were only somewhat better at estimating the diets based on the inner blubber FA signatures (55.3% error) (Table S6). The CCs from these other species overestimated the proportion of herring (76.1% herring on average) and underestimated the proportion of capelin (16.6% on average) in the diet of the managed-care whales, especially when used on the full blubber and outer blubber FA signatures. Thus, we chose to only use CCs derived from killer whales for remainder of the study.

**Figure 3:**
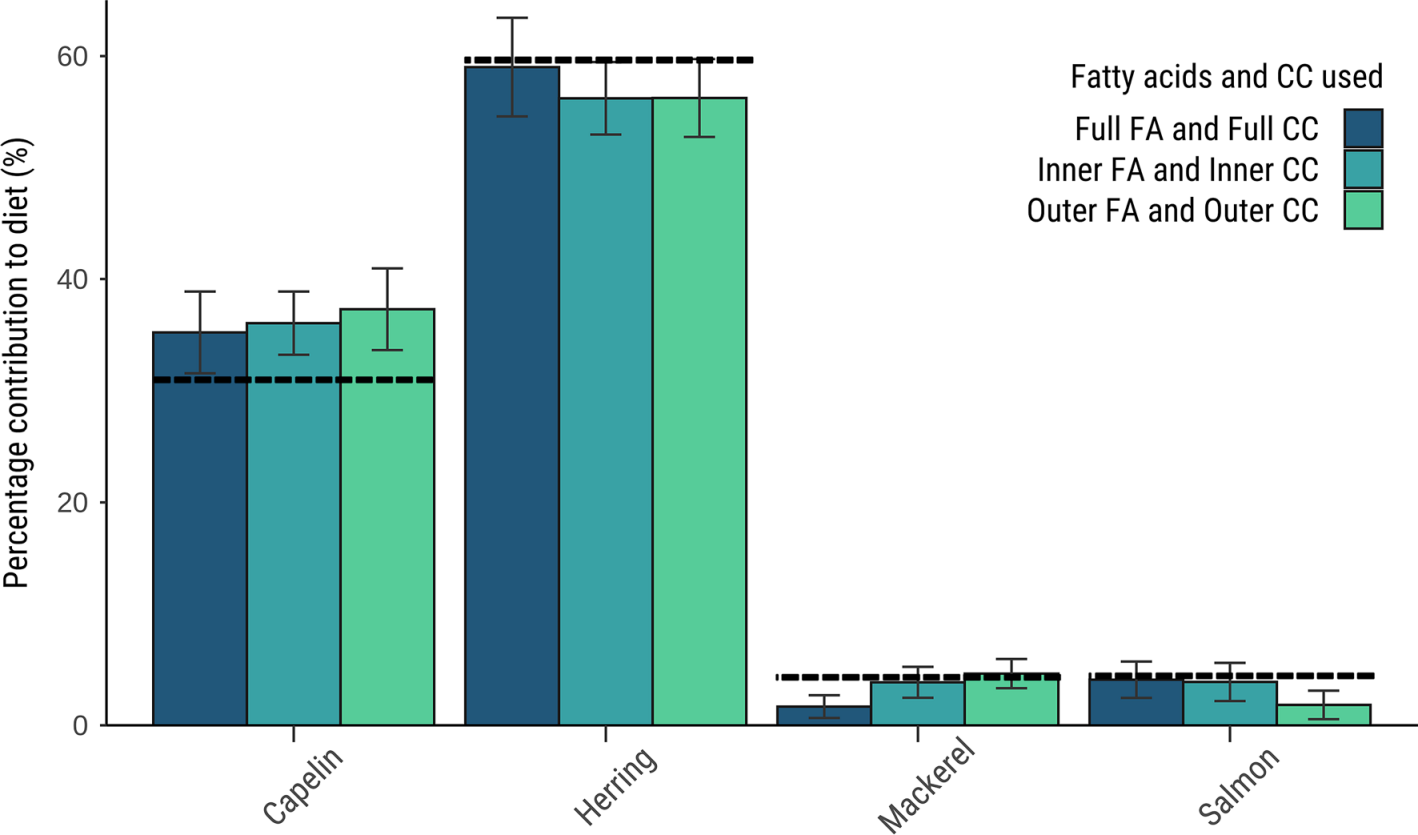
Mean diet estimates (%) for the four managed-care killer whales obtained for the cross-validation analyses (estimating the diet of two whales using the CCs of the two other whales) and based on the prey library consisting of capelin (n = 10), herring (n = 10), mackerel (n = 10) and salmon (n = 4). The Kullback–Leibler (KL) distance was used with the dietary FA set. The true diet (dash line) fed to the managed-care killer whales consisted of 32% capelin, 60% herring, 4% mackerel and 4% salmon.

Diet estimates showed good accuracy in the cross-validation tests. (*i.e*.: running the model on two of the four managed-care killer whales, using the average CCs from the two other whales, thus running six different simulations per layer; Table S7). The average diet estimates were accurate (18.0% error with the KL distance, and 25.1% error with the Aitchison distance), with estimates for capelin and herring being very close to the true diet: 35.2 ± 7.33 (vs. 32%) for capelin and 59.1 ± 8.9 (vs. 60%) for herring (KL distance). Two-by-two comparisons for the inner blubber yielded accurate estimates (6.3% error with the KL distance vs. 39.6% with the Aitchison distance). Similarly, two-by-two comparisons for the outer blubber resulted in more accurate estimates with the KL distance (23.3% error with the KL distance vs. 30.2% with the Aitchison distance; Fig. 3, Table S7).

Although both statistical distances resulted in accurate diet estimates, the KL distance was selected as it resulted in more accurate estimates than the Aitchison distance when used with killer whale CCs. Additionally, diet estimates computed using the KL and CC means from the four whales were closer to the true diet than when using the Aitchison distance: 21.7% error (KL) vs. 22.1% (Aitchison) for full blubber FA signatures with full blubber CCs; 16.8% error vs. 29.8% error for the inner blubber FA signatures with inner blubber CCs; and 25.7% error vs. 32.1% error for outer blubber FA signatures with outer layer CCs (Tables S8 and S9). Both the Aitchison and KL distances scored a high correct attribution rate of the prey to its species (an average of 91.0% for the Aitchison distance vs. 84.4% for the KL distance), and results were too close to determine which LOPO analysis performed better (Table S4).

The diet of the managed-care killer whales was accurately estimated using killer whale CCs, provided that the appropriate layer-specific CC was used (e.g., inner blubber CCs to model the diet based on inner blubber FA signatures; Figs. 3 and S3). The accuracy was lower when we did not use the CC set corresponding to the FA layer (Tables S8 and S9). Indeed, while using full blubber CCs on full blubber FA signatures resulted in 21.7% error compared to the true diet, using full blubber CCs on outer blubber FA signatures resulted in 86.0% error with a large overestimation of herring (89.6% estimated vs. the true diet of 60%). Our results with the KL distance showed that the inner layer average CCs yielded more accurate estimates (16.8% error) on inner blubber FA signatures compared to layer 1 CCs (50.7% error). In the same way, layer 1 CCs produced the most accurate estimates when used on layer 1 FA signatures (18.1% error vs. 48.4% error on inner blubber FA signatures), and layer 10 CCs yielded more accurate estimates when used on layer 10 FA signatures (20.2% error) than when used on outer blubber FA signatures (67.8% error). Finally, outer blubber CCs yielded good accuracy when used on the outer blubber FA signatures and on layer 10 FA signatures (25.7% error for outer blubber FA, and 33.5% error for layer 10 FA).

### Diet estimations in the free-ranging killer whales and application of the model to remote biopsies

With the determination of the dietary FA set, the KL distance, and the layer-specific killer whale CCs being the optimal parameters for estimating managed-care killer whale diets, we applied QFASA to estimate the diets of free-ranging killer whales from Greenland and the Faroe Islands. Since these killer whales were harvested or stranded, we had access to full blubber samples and were able to estimate the diets in a layer-specific manner. First, we verified our prey-library by running the LOPO analysis on the prey library and found that harp seals (*Pagophilus groenlandicus*) and hooded seals (*Cystophora cristata*) overlapped. Therefore, harp seals and hooded seals were grouped into one category for diet estimation purposes. Additional QFASA method checks on the free-ranging killer whales (including the justification for using the KL distance over the Aitchison distance) are available in the supplementary text, and Table S10.

**Figure 4:**
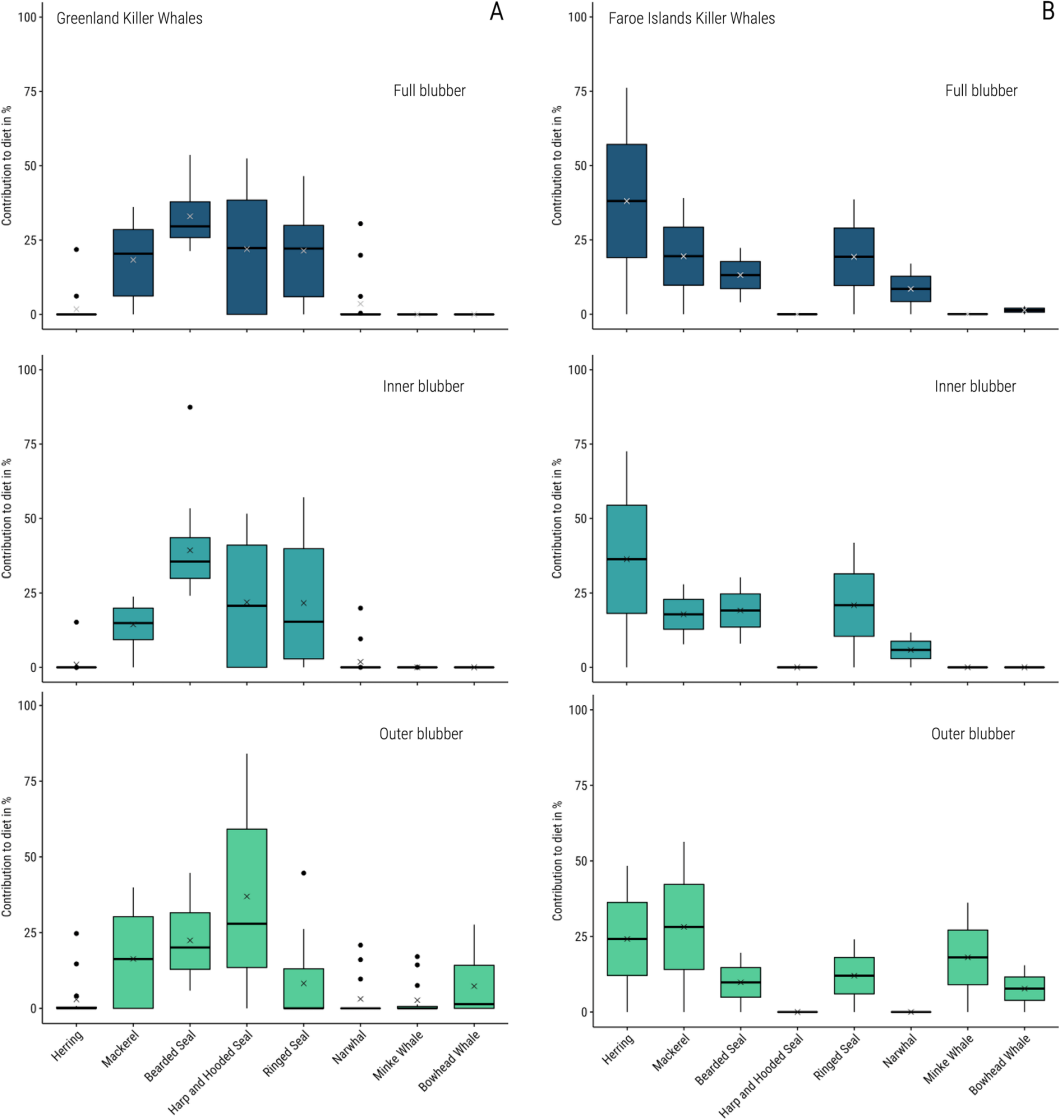
Proportions of different prey species estimated in the diets of (**A**) Greenland (n = 16) and (**B**) Faroe Islands (n = 2) killer whales based on the validated quantitative fatty acid signature analysis (QFASA) approach for killer whales. For outer layer, inner layer, and full depth FA signatures, outer layer, inner layer, and full depth calibration coefficients (CCs) were used, respectively. The estimates were lipid-corrected to account for differences in lipid between the prey items. The crosses show the mean, the thick bar shows the median, and the box extremities show the lower and upper quartiles.

Based on our dietary estimates, free-ranging killer whales in Greenland (n = 16) fed mainly on all species of seals present in our prey library, as well as on mackerel (Fig. 4). We ran QFASA models separately on the full depth, inner blubber, and outer blubber FA signatures, using the full depth CCs, inner blubber CCs and outer blubber CCs, respectively. The proportion of total seals in the Greenlandic killer whales’ diets was estimated to be 82.6 ± 5.9% in the inner blubber, 67.0 ± 5.2% in the outer blubber and 75.9 ± 4.7% in the full blubber, and consisted of bearded seal (*Erignatus barbatus*), harp/hooded seal, and ringed seal (*Pusa hispida*) (Fig. 4). The proportion of total fish was estimated at 20.0 ± 3.4% in the inner blubber, 15.5 ± 1.6% in the outer blubber and 20.4 ± 2.4% in the full blubber. This consisted nearly entirely of mackerel (*Scomber scombrus*), with almost no herring (*Clupea harengus*) estimated in the diets. Diet estimates were low for baleen whales in all layers except to some extent in the outer blubber, where bowhead whales were estimated to minimally contribute to the killer whales’ diet (7.0 ± 2.5%). We reported the individual estimates for the inner layer for each free-ranging killer whale in Table S11 with stomach content data, when available. Killer whales with harp and/or hooded seals reported in their stomachs often had a high diet estimate for harp and hooded seals, and for seals in general (between 51.7% and 100% for total seal percentage). Conversely, both killer whales from the Faroe Islands (n = 2) showed higher proportions of fish (herring and mackerel) in their dietary estimates (27.9% and 80.3%) than all the Greenland whales. One of the two Faroese killer whales was estimated to have fed nearly exclusively (72.6%) on herring, while the other whale had an estimate of mackerel (27.9%) and ringed and bearded seals (Fig. 4).

**Figure 5:**
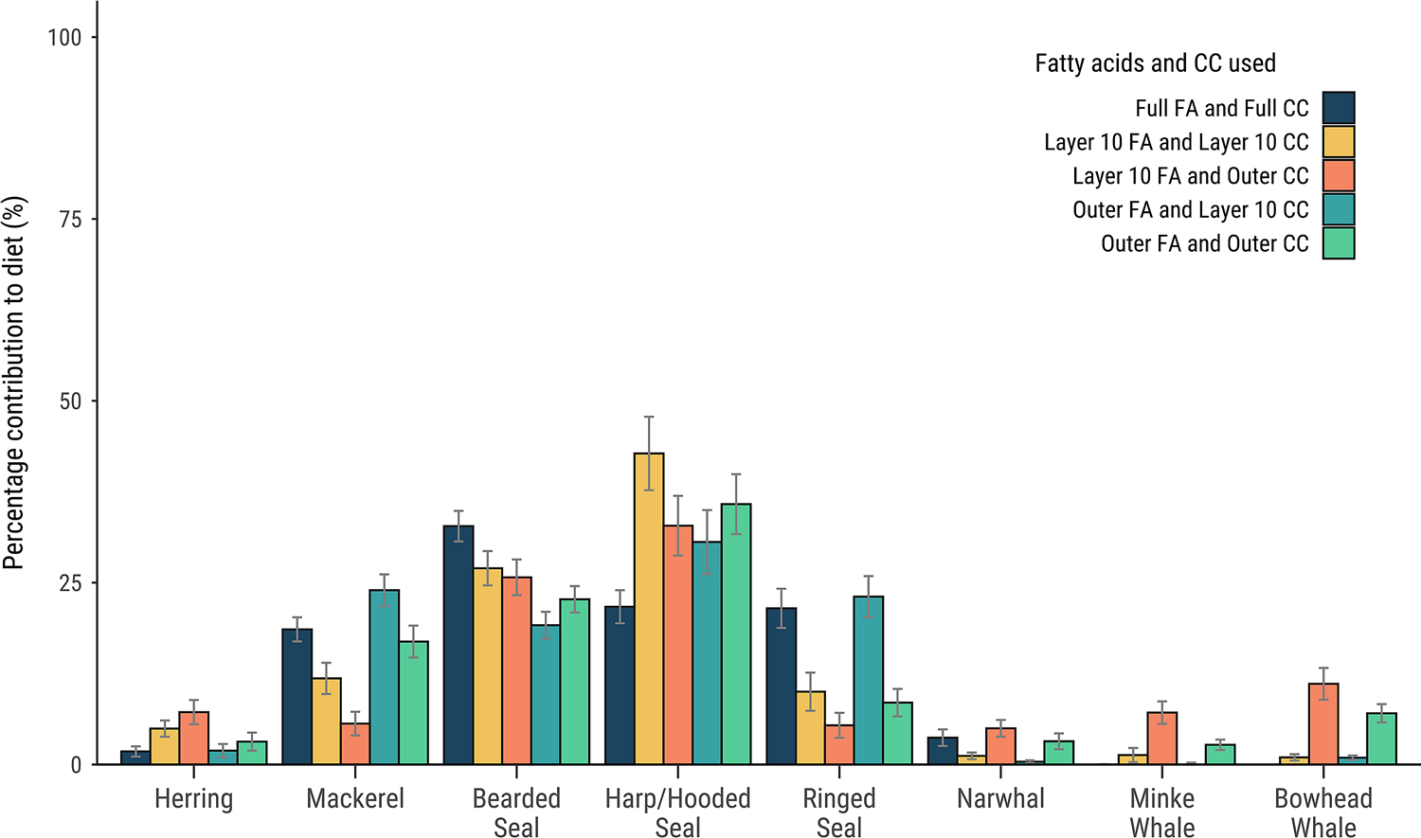
Diet estimates for Greenlandic killer whales (n = 16) for full blubber FA signatures and full blubber CCs, and four combinations of outer blubber and layer 10 (the outermost layer) FA signatures and CCs. The simulated diets resulted in a similar percentage of prey species, with the four main species being harp and hooded seal, mackerel, bearded seal, and ringed seal.

Remote biopsies from free-ranging cetaceans typically only collect the outer blubber layers, e.g., layer 10 and likely layers 9, 8, 7 and/or 6. To test whether the newly developed QFASA model for killer whales could accurately predict the diet of free-ranging individuals from biopsy samples, we ran QFASA on the outer blubber and layer 10 FA signatures using the outer blubber CCs or layer 10 CCs (four combinations total) and found the estimates were similar, and close to the diet estimates using full blubber FAs signatures and full blubber CCs (Fig. 5), with the four main prey species being harp and hooded seal, mackerel, bearded seal, and ringed seal.

## Discussion

Our results show that with the appropriate CCs, the diets of killer whales can be accurately estimated using QFASA. To use this approach, blubber-layer-specific CCs are essential; we demonstrated this for killer whales, but it is likely also the case for other odontocetes and possibly other cetaceans. Using no CCs resulted in highly inaccurate estimates for the managed-care whales, based on their known diet. Mustelid and pinniped CCs also did not produce accurate estimates for the managed-care killer whales. These findings were perhaps not surprising, given differences in metabolic characteristics between the order Artiodactyla (even-toed ungulates, which includes cetaceans) and Carnivora (which includes pinnipeds and mustelids)^25^, as well as the high degree of stratification of FAs in cetacean blubber compared to pinniped blubber or mustelid adipose tissue^10,14,21^.

We were able to calculate and validate CCs for full depth blubber, as well as for inner and outer blubber. Our cross-validation simulations showed a high average accuracy using the full blubber CCs and full blubber FA signatures. Thus, when full blubber FA signatures are available, such as for stranded or harvested cetaceans^26,27^, full blubber CCs should be used. However, for most free-ranging cetaceans, only small biopsies containing skin and partial (outer) blubber depth profiles are typically available. Due to the FA stratification through blubber depths, we found that the full blubber CCs do not provide reliable diet estimates for partial depth blubber FA signatures. Critically, we were able to overcome this issue to accurately predict the diets of managed-care individuals by generating and applying layer-specific CCs. Using the layer ten (the outermost layer) or the outer blubber (layers 6–10) CCs, with either the layer 10 or outer blubber FA signatures, produced similar dietary estimates in the free-ranging killer whales, which could be explained by a minor difference in the FA signatures within the outer layers (6–10) in these individuals^10^. Nonetheless, outer blubber CCs yielded more accurate estimates for the managed-care killer whales when applied to either outer blubber or layer 10 FAs, perhaps because the outer blubber CCs account for potential small differences between the layers in the outer blubber. Therefore, we recommend using outer blubber CCs to estimate the diet of cetaceans from biopsy-derived FA signatures. Although layer 1 CCs did not perform as well as the inner blubber CCs on the inner blubber FA signatures, which can be explained by FA signature varying slightly from layer 1 to 4 (Fig. S1, Bourque et al. 2018), the diet estimates were accurate when using the average inner layer CCs on either layer 1 or inner blubber FA signatures. Therefore, if researchers focus on using inner blubber FA signatures to model recent blubber deposition for example, we recommend using the inner blubber CCs for more accurate dietary estimations.

In addition to validating QFASA for killer whales based on comparison of the managed-care killer whale diet estimates to their true diet, further support for the approach came from the consistency in the diet estimates in free-ranging killer whales with stomach contents and available literature on their feeding habits. The QFASA method, using full, inner, and outer blubber, estimated harp/hooded seals as one of the top three diet items for Greenlandic killer whales, consistent with their stomach contents. Stomach contents were reported for seven killer whales, and all included harp and/or hooded seal remains. Killer whales in West Greenland waters have also been reported to feed heavily on marine mammals based on visual observations^28^. Nonetheless, the LOPO analysis revealed that only 58% of harp and/or hooded seals were identified correctly, with some overlap with ringed seals, thus the species-specific seal consumption estimates may be less robust than for the other prey items.

In addition to seals, Atlantic mackerel were also estimated to be an important part of killer whale diets in Greenland killer whales; while almost no predation on herring was estimated. Observations in Norway have shown an increasing association between killer whales that forage offshore and mackerel^29–31^ and an increase in mackerel biomass has been reported in the Irminger Current, off Tasiilaq, where the Greenlandic killer whales were harvested^32^. Increases in mackerel in Greenland may be explained by the warming temperatures in the Arctic, particularly in East Greenland, which has changed the migrating pattern of mackerel from the Norwegian and North Seas towards Greenland over the past decade^32^. Previous studies suggested that some populations of North Atlantic killer whales are strongly associated with mackerel stocks^7^. Since killer whales are opportunistic hunters capable of switching prey^6^, one could easily imagine killer whales in Greenland having a mixed diet of both mackerel and seal species.

Unlike seals and fish, almost no consumption of the three whale species in our library was estimated by QFASA, except for some consumption of narwhal (*Monodon monoceros)* (9.6–19.9%) for two individuals. One of these individuals was also the only killer whale to have whale reported in their stomach contents, although the identified species was minke whale (*Balaenoptera acutorostrata*). The only other exception was for bowhead whale (*Balaena mysticetus*) estimates in the outer blubber of the Greenlandic killer whales, which could indicate that bowheads are part of an occasional feeding. However, local reports off Tasiilaq suggested that killer whales occasionally prey on humpback whales or fin whales (Rosing-Asvid, pers. comm.). We attempted to include humpback whales in our analysis but had to drop them because their FA signatures overlapped with the other prey signatures (Supplementary text). The higher proportion of bowhead whales in the outer blubber could indicate occasional feeding on some species of baleen whales in Eastern Greenland, although the estimates are still quite low compared to the other species of fish and seal. Thus, both short-term (stomach contents) and longer-term (QFASA) estimates were similar in suggesting a limited importance of cetacean species in the diets of these killer whales in Greenland. This feeding appears to differ from killer whales feeding in other Arctic environments; specifically, the killer whales in West Greenland-eastern Canadian Arctic are known to target narwhals and other whales including bowheads^33–35^.

The two killer whales from the Faroe Islands were estimated to have different diets from the Greenland killer whales based on QFASA. One was estimated to eat mainly herring, which aligns with reports stating that herring is the preferred prey of killer whales in the Faroe Islands^36^. The other had a high proportion of mackerel, but also ringed and bearded seals in its diet estimates. Both Faroese whales were expected to have high proportions of fish in their diet estimates, given the lower concentrations of biomagnifying contaminants in their blubber, compared the Greenlandic whales^37^. The presence of the two Arctic seal species in this whale’s diet seems unlikely and may reflect another type of pinniped prey of some Faroese, Icelandic, and Norwegian killer whales, like grey or harbor seals^36,38,39^. The prey library available for this study did not include grey and/or and harbor seals, but future work could likely better identify seal species by including more appropriate additional seal prey in the library.

Future research should investigate the use of QFASA in other odontocetes and cetacean species to shed light on inter-and intra-species and -population differences in the application of QFASA methods and in elucidating dietary variations. Across odontocetes, stratification indices (SI), which represent the differences in concentration of the 16 predominant FAs in the outer vs. inner blubber, have been calculated as the summed absolute values of the outer vs. inner blubber differences^21^. From these data, it seems likely that our QFASA approach could be applied to other odontocetes with stratification indices similar to killer whales (SI = 31.4), which includes for example pilot whales (*Globicephala melas*), beluga whales, and narwhals^21^. To use QFASA on odontocetes or other cetaceans with unknown stratification indices, samples obtained from stranded animals can be used to calculate the SI for the species. If a significant difference in stratification were found, the method would likely need to be adjusted according to the number and depth of layers in the blubber.

This study demonstrates the utility of QFASA in estimating the diets of killer whales and likely other cetaceans, including the use of remote biopsy samples. Nonetheless, accurate diet estimates using this approach require certain conditions be met. In particular, QFASA relies on a complete prey library^40^. While our prey library contained prey species that are believed to be part of the Greenlandic killer whales’ diets, some potentially important prey species could be missing from the library. For example, squid has occasionally been reported in the whales’ stomachs^28^, but was not included in the current library. The stomach contents of a killer whale harvested in September 2021 also included redfish (*Sebastes marinus*), Greenland halibut (*Reinhardtius hippoglossoides*), and Atlantic cod (*Gadus morhua*) (Rosing-Asvid, pers. comm.). Our results showing Arctic seals in the diet estimates of one Faroese whale seem unrealistic and could have represented another species of pinniped(s) with similar signatures. Thus, a carefully conceived prey library, based on prior feeding knowledge, is an important consideration. Additionally, while we used the FA signatures from the blubber of the marine mammal prey (which likely represent most of the body’s lipid content), other parts or whole bodies^41^ may be consumed, which may have somewhat different FA signatures or lipid content than the full body lipid percentage we used. Finally, while previous studies have shown that FAs deposit in the inner blubber within a couple of weeks, we cannot yet estimate how long it may take for dietary FAs to deposit within the outer blubber. Our managed-care individuals were fed a constant diet over many years, thus not enabling us to determine an exact time frame for outer blubber FA deposition. With these key considerations in mind, this new QFASA approach should nevertheless provide important new insight into the feeding ecology of free-ranging killer whales and other cetaceans.

## Methods

Full depth blubber samples were collected and analyzed from previously deceased managed-care (n = 4) and subsistence-harvested free-ranging (n = 18) killer whales, and the samples were divided into ten equal length sections as described in Bourque et al., 2018. Prey from the known constant long-term diet of the managed-care whales (n=34) and potential prey of free-ranging whales (n=535) were also collected and analyzed. Detailed information regarding all sample collections and FA analyses can be found in the supplementary text and Tables S1 and S2.

### Development of QFASA using managed-care whales

In addition to FA signatures of the predator and of all potential major prey, the QFASA model requires (1) designating a particular set of FAs to use from the ~ 70 routinely monitored, (2) the development of species-specific CCs, (3) the relative fat content of prey items, and (4) a statistical model that estimates the proportional prey composition by minimizing the statistical distance between the CC-corrected predator FA signatures and the average prey FA signatures^14^. The approach for each of these steps is detailed below.

#### Fatty acid sets

To develop the model, we tested two sets of FAs, dietary FAs, which only arise from the diet, and extended dietary FAs that also include FAs partially biosynthesized by the predator^14^. The first set included every dietary FA^14^ that was greater than 0.1% of the total FA signature to minimize analytical variation associated with low level FAs; this set consisted of 21 FAs (Table S2). The second set included all extended dietary FA^14^, but again only those exceeding 0.1% of total FAs; this second set contained 30 FAs.

#### Calibration coefficients

The CCs were generated from the FA signatures of the four manage-cared killer whales and the FA signatures from the four species that comprised their constant, long-term (multiple years) diets. As Bourque et al. (2018) found no statistically significant differences between layers 1–4 and layers 6–10 for the killer whale samples, throughout the current study, “full blubber FA” refers to the lipid-weighted average of all 10 layers, while “inner blubber FA” refers to the lipid-weighted average of layers 1–4 and “outer blubber FA” refers to the lipid-weighted average of layers 6–10. Three CC sets were estimated using the lipid-weighted FA signatures averaged across layers for: full blubber CCs, inner blubber CCs, and outer blubber CCs. The CCs were calculated as the ratio of a given FA in each blubber layer of a killer whale to the ratio of that FA in its diet, weighted by the proportions of each diet item, as follows

$${CC}_{{FA}_{i,{KW}_{j}}}=\frac{{FA}_{i,{KW}_{j}}}{\left({FA}_{i}, {Herring}_{j}\right)\times 0.6+\left({FA}_{i}, {Capelin}_{j}\right)\times 0.32+\left({FA}_{i}, {Mackerel}_{j}\right)\times 0.04+ \left({FA}_{i}, {Salmon}_{j}\right)\times 0.04}$$

where FA_i_ is the percent of a FA *i* in the whale or prey item *j*, and 0.60, 0.32, 0.04, and 0.04 in the denominator are the weight fractions of the respective prey in the whales’ diets. Given that there were four killer whales, ten capelin *(Mallotus villosus)*, ten Pacific herring (*Clupea pallasii)*, ten Pacific mackerel (*Scomber japonicus*), and four sockeye salmon (*Oncorhynchus nerka*), we ran all possible combinations of killer whales and diet items, which generated 4000 CCs per FA per killer whale for each blubber layer. From this, we calculated the 10% trimmed mean for each killer whale. The final CC for each blubber layer was computed as the mean of the four trimmed means. As with FAs, full blubber CCs correspond to the average of the ten layers, while the inner blubber CCs correspond to layers 1–4 and outer blubber CCs correspond to layers 6–10, all consisting of FA signatures of the killer whale weighed by the lipid content of each layer. In addition to using our own killer whale-derived CCs and to determine the sensitivity of the method to different CC sets, we also ran QFASA with previously published mink CCs (herring-fed) and grey seal CCs^14,18^, as they have been the most used CCs in other QFASA studies on marine mammals. The list of CCs used in this study can be found in Table S3.

#### The QFASA statistical method

The QFASA model was run in R, version 3.6.1. (R Core Development Team 2019) using the QFASAR package^42^. Diet estimations in QFASA rely on multiple assumptions. First, QFASA relies on the principle that predator FA signatures can be modeled as a linear mixture of the prey FA signatures^14^. Thus, we expect the predator FA signature to be within the prey FA range; and not meeting these criteria indicates poor CCs or an incomplete prey library^42^. We tested our data using the function *pred_beyond_prey* in QFASAR to estimate the proportion of the predator’s FA values that were outside the range of their prey values. To visually test for the improvement of the datasets when full blubber CCs were applied to the predator full blubber FA signatures, we also performed a Principal Component Analysis (PCA) using the FactomineR package with the FAs (both with and without CCs applied) and the prey signature to visualize whether use of the CCs brought the FA signatures of the predator closer to the prey FA space. A second QFASA assumption is limited overlap among the prey species’ FA signatures^14^. To test for this assumption, we used the *leave_one_prey_out* (LOPO) function which removes one prey signature from the library at a time and recomputes the mean prey-type and then estimates the diet of the removed prey signature. The analysis performs this computation on each prey signature, one at a time. The final output indicates the proportion of samples attributed to the correct species. We chose the best FA set to use based on their performance in the LOPO and *pred_beyond_prey* analyses.

After choosing the best FA set, we ran multiple simulations using different sets of CCs. Each set of simulations was run using both the Kullback–Leibler (KL) distance^14^ and the Aitchison distance^43^, as the literature has not yet settled on the best distance to use^44^. To evaluate which distance performed best, we determined how both scored in both the QFASA diagnostics (assumptions tests) and on the accuracy of the diet estimates relative to the true diet of the managed-care killer whales.

First, to quantitatively test the need for CCs, we ran QFASA without CCs since some studies have suggested that QFASA might not need CCs to provide correct estimates^45,46^. Two simulations were run on full blubber FAs with no CCs: one with KL distance and the other with Aitchison distance. Each simulation resulted in different proportions of prey species in the diet, referred to hereafter as “diet estimates”. The means and SEs for the diet estimates were computed using bootstrap sampling (n = 100), as previously described^23,47,48^. The accuracy of each diet estimate was inferred from the % error (absolute value (true diet estimate—modeled diet estimate)/ true diet estimate *100) to assess whether accurate diet estimates could be achieved without the use of CCs. Next, we compared the accuracies of the diet estimates using CCs developed for phocids and mustelids on different FA layers to assess the need for a cetacean-specific CC set^14,18^. Three simulations were run on the dietary FA set with either mink or grey seal CCs using the KL distance (on full blubber, inner blubber, and outer blubber) and three on the same CCs using the Aitchison distance. We then tested the accuracy of the diet estimates using the CCs we developed from the managed-care whales and their diets. Here, it would have been circular to estimate the diets of the same killer whales that were used to generate the CCs; instead, we cross-validated the CCs, by estimating the diet of two of the four killer whales (either full, inner, or outer blubber FA signatures) using the mean full, inner or outer blubber CCs of the two other killer whales. We performed this for all possible combinations of killer whales (six) for each part of the blubber. We then determined the mean diet estimates generated from these iterative analyses and calculated the % error relative to the true diet. This allowed us to test the robustness of these CCs and ensure that the individual CC variation did not impact the overall diet estimates across the layers. Next, we ran simulations with blubber layer-specific CCs (the average of the four whales for each layer) and layer-specific FA signatures to test the need for layer-specific CCs. To do this, we ran twenty simulations in total on the managed-care killer whales (ten with the KL distance, and ten with the Aitchison distance). Out of the ten simulations for each distance, five matched the CC layer to the FA layer, testing the accuracy of the diets, and five were mismatched, testing whether matching CCs to their respective FA mattered, and especially for the outer layers representing a biopsy to see which CC set worked best on these outer blubber FA. The options were: (1) full blubber FA and full blubber CCs, (2) layer 1 FAs and layer 1 CCs, (3) layer 1 FAs and inner blubber CCs, (4) inner blubber FAs and layer 1 CCs, (5) inner blubber FAs and inner blubber CCs, (6) outer blubber FAs and full blubber CCs, (7) outer blubber FAs and outer blubber CCs, (8) outer blubber FAs and layer 10 CCs, (9) layer 10 FAs and outer blubber CCs and (10) layer 10 FAs and layer 10 CCs.

### Diet estimations in the free-ranging killer whales and application of the model to remote biopsies

After choosing the FA sets, CC sets and statistical distance that yielded the most accurate estimates, we applied QFASA to the free-ranging killer whales using our prey library consisting of various fish and marine mammal species. Due to the large difference in fat percentage between the potential prey types (marine mammal vs fish), we adjusted the dietary estimates to prey lipid percentages using the *adj_diet_fat* function. For fish, we used the whole-body fat % calculated during the lipid extraction (mean: 16% for herring, 22% for mackerel). Because killer whales generally do not just eat the blubber when they consume marine mammals, we used a fat percentage of 30%, which was the average fat percentage calculated for the whole body of harbor seals^49^. Given the lack of whole-body fat percentage for other marine mammal species we also used 30% for whales as prey.

Remote biopsies from free-ranging cetaceans (usually between 10 and 40 mm depth^50^) typically only collect the outer blubber layers , e.g., some combination of layers 10 through possibly 6, as defined in our analysis. To confirm that the newly developed QFASA model on killer whales could accurately predict diets of free-ranging individuals, and to determine which CCs should be used on biopsies, we ran QFASA on the outer layers (6–10) and layer 10 FA using the outer layer CCs or layer 10 CCs (four combinations total) and compared the resulting estimates with the ones generated using full blubber FAs and full blubber CCs.

## Supplementary Information

Supplementary Information.

## Data Availability

Most of our data came from published papers as clearly stated in our sample and collection details (Table S1); some of the prey data for Greenland (16 narwhals and five minke whales) is unpublished and will be published within the next two years by other members of our lab. We have uploaded the fish data from SeaWorld and Greenland to https://www.polardata.ca/. The data from the 16 narwhals and five minke whales will be uploaded on the same public database when our lab members publish the papers using them. We included the calibration coefficients (CCs) we generated in Table S3, and the other CCs came from https://figshare.com/articles/dataset/Appendix_D_A_table_and_figure_presenting_calibration_coefficients_for_polar_bears_derived_from_captive_feeding_studies_on_mink_Mustela_vison_/3566526 and https://esajournals.onlinelibrary.wiley.com/cms/asset/8b50fe0e-98e8-4089-a178-6ffde2ebc5bc/ecm2004742211-tbl-0001-m.jpg. Additional CCs calculated for each layer are available upon request.
