# Supplementary material for: Validation of quantitative fatty acid signature analysis for estimating the diet composition of free-ranging killer whales
Source: Sci Rep. 2022 May 13;12:7938. doi: 10.1038/s41598-022-11660-4 (PMC9106655; doi:10.1038/s41598-022-11660-4)
Supplement: Supplementary file 1 — Supplementary Information. [file 41598_2022_11660_MOESM1_ESM.docx]

**Supplementary Information**

**Validation of quantitative fatty acid signature analysis for estimating the diet composition of free-ranging cetaceans**

Anaïs Remili ^1^*, Rune Dietz ^2^, Christian Sonne ^2^, Sara J. Iverson ^3^, Denis Roy ^1^, Aqqalu Rosing-Asvid ^4^, Haley Land-Miller ^1^, Adam F. Pedersen ^1^, Melissa A. McKinney ^1^*.

**Affiliations:**

^1^ Department of Natural Resource Sciences, McGill University, Sainte-Anne-de-Bellevue, QC H9X 3V9, Canada

^2^ Department of Bioscience, Arctic Research Centre, Aarhus University, Roskilde DK-4000, Denmark

^3^ Department of Biology, Dalhousie University, Halifax, NS, Canada

^4^ Greenland Institute of Natural Resources, Nuuk 3900, Greenland

*Corresponding author: anais.remili@mail.mcgill.ca, melissa.mckinney@mcgill.ca

**List of Tables**

Table S1: List of sample and collection details for the managed-care killer whales and their prey, as well as the free-ranging killer whales and their potential prey.

Table S2: Fatty acid (FA) percentages (mean ± SE) in the managed-care killer whales (n=4) and their prey items. Only FA greater than 0.1% are shown. The prey species were: Pacific herring (*Clupea pallasii)*, capelin *(Mallotus villosus)*, Pacific mackerel *(Scomber japonicus)*, and pink salmon *(Oncorhynchus gorbuscha).* Italicized FAs are the extended dietary set and bold FAs are the dietary set. Normal font represents non-dietary FAs (which were not included in the QFASA analyses).

Table S3: Calibration coefficients (CCs) used in the QFASA simulations. Killer whale CCs were calculated for the managed-care killer whales from this study. CCs showed here are for the dietary (bold) and extended dietary FAs that were greater than 0.1% of total FAs. Layer 1 is closest to muscle, while layer 10 is closest to skin.

Table S4: Leave-one-prey-out (LOPO) simulations on the prey library of the managed-care killer whales to establish a potential overlap of prey FA signatures. Numbers in bold represent the percentage of prey correctly identified.

Table S5: QFASA diet estimates for managed-care killer whales (n=4) using the full blubber FA signatures and no CCs. The managed care killer whales’ actual diet consisted of 32% capelin, 60% herring, 4% mackerel and 4% salmon.

Table S6: QFASA diet estimates for managed-care killer whales (n=4) using marine-fed mink and grey seal CC. Simulations resulted in inaccurate estimates compared to the killer whales’ actual diet which consisted of 32% capelin, 60% herring, 4% mackerel and 4% salmon. The mean and SE were bootstrapped 100 times.

Table S7: Two-by-two comparison of the diet estimates (in %) using the FAs from two managed-care killer whales and the CCs from the other two remaining killer whales (n=4 total) on the inner and outer blubber FA signatures and the inner and outer blubber CCs.

Table S8: QFASA diet estimates for managed-care killer whales (n=4) layer-specific CCs derived from the same managed-care killer whales with the Aitchison distance. Diet estimates were more consistent and accurate with the Aitchison distance compared to the Kullback-Leibler (KL) distance. The managed-care killer whales’ actual diet consisted of 32% capelin, 60% herring, 4% mackerel and 4% salmon. The mean and SE were bootstrapped 100 times. Layer 1 is closest to muscle, while layer 10 is closest to skin.

Table S9: QFASA diet estimates for managed-care killer whales (n=4) layer-specific CCs derived from the same managed-care killer whales with the KL distance. Diet estimates were more consistent and accurate with the Aitchison distance compared to the KL distance. The managed-care killer whales’ actual diet consisted of 32% capelin, 60% herring, 4% mackerel and 4% salmon. The mean and SE were bootstrapped 100 times. Layer 1 is closest to muscle, while layer 10 is closest to skin.

Table S10: Leave-one-prey-out (LOPO) analysis of the Greenlandic prey library using the Aitchison distance and the KL distance on the dietary FA set.

Table S11: Intra-population variation in QFASA estimates for East Greenland killer whale, and relevance of estimates regarding existing stomach content data.

**List of Figures**

Figure S1: Representation of the blubber layers in our killer whale samples, including full blubber, outer and inner blubber, as well as the typical depth of a biopsy.

Figure S2: Blubber depth variation in the FA CCs calculated from the managed-care killer whales and their known diet items for the dietary (with asterisk) and extended FAs greater than 0.1% of total FAs. Layer 1 is closest to muscle, while layer 10 is closest to skin.

Figure S3: Mean diet estimates (in %) for the four managed-care killer whales when using the average CCs for the four individuals, based on the prey library consisting of capelin (n = 10), herring (n = 10), mackerel (n = 10) and salmon (n = 4). The Kullback-Leibler (KL) distance was used with the dietary FA set. The true diet (dash line) fed to the managed-care killer whales consisted of 32% capelin, 60% herring, 4% mackerel and 4% salmon.

Figure S4: Principal component analysis of dietary FA signatures in prey and Greenlandic killer whales (full blubber) without CCs (in orange) and with CCs (full blubber) generated for this study (in red). Applying CCs to the predator puts them closer to the range of prey FA.

Additional Methods

Samples from managed-care and free-ranging killer whales and their prey

For the managed-care killer whales, we used existing FA signature data from archived blubber samples of four previously deceased (one in 2008 and three in 2010) individuals from SeaWorld ^1^. None of the individuals suffered from pre-terminal weight loss, which could lead to differential mobilization and metabolism of individual fatty acids ^1^. The killer whale samples were frozen (−20 °C) after collection and during transport and stored at −80 °C from August 2015 until analysis in November 2015 through March 2016. Samples from these individuals were in the form of full depth blubber pieces with skin attached. The blubber was divided into ten equal-length pieces, from adjacent to muscle (layer 1) to adjacent to skin (layer 10). The managed-care killer whales had been fed a long term (over multiple years) diet consisting of roughly constant proportions of Pacific herring (*Clupea pallasii*, 60%*)*, capelin *(Mallotus villosus*, 32%), Pacific mackerel *(Scomber japonicus*, 4%*)*, and sockeye salmon *(Oncorhynchus nerka*, 4%*)*. To create the appropriate prey library, SeaWorld provided frozen whole fish samples from the supply they fed to the killer whales (herring, n=10; capelin, n=10; mackerel, n=10; and salmon, n=4). Fish samples were received in May and June 2016 and analyzed for fat content and FA composition in Sept 2016 through March 2017.

We used published FA signature data from 18 subsistence-harvested free-ranging killer whales, from Greenland (n =16) and the Faroe Islands (n = 2) (Bourque et al., 2018) to test the QFASA model on wild individuals. These samples were stored frozen (−20 °C) after collection in 2008-2014 and during transport and at −80 °C once received in May 2015. The samples were analyzed in Nov 2015 to July 2016. The full depth blubber pieces with skin attached were divided into 10 equal length sections, exactly as per the managed-care individuals. The prey FA library consisted of 535 samples from nine different species: Atlantic herring (*Clupea harengus*, n = 10*)*, Atlantic mackerel (*Scomber scombrus*, n = 10), bearded seals (*Erignathus barbatus*, n = 55), harp seals (*Pagophilus groenlandicus*, n = 239), hooded seals (*Cystophora cristata*, n = 32), ringed seals (*Pusa hispida*, n = 106), narwhals (*Monodon monoceros*, n = 16), bowhead whales (*Balaena mysticetus*, n = 62) and minke whales (*Balaenoptera acutorostrata*, n = 5). The bearded, harp, hooded, and ringed seal samples were from Greenland and the Davis strait and were published previously ^2-4^. The bowhead whales were from Alaska, with FA data provided by Dr. Suzanne Budge ^5^. The Atlantic herring, Atlantic mackerel, narwhal and minke whale samples were from Greenland and the FA data were generated as part of the current study. Herring and mackerel were received with the Greenland killer whale samples and analyzed along with the SeaWorld fish from Sept 2016 to March 2017. Collection details for all managed-care and free-ranging killer whales and prey samples are provided in Table S1.

Table S1: List of sample and collection details for the managed-care killer whales and their prey, as well as the free-ranging killer whales and their potential prey.

| Species | ID | Geography | Date | Paper originally published |
| --- | --- | --- | --- | --- |
| Killer Whale | 35143 | East Greenland | Summer 2013 | Bourque et al. 2018 |
| Killer Whale | 38340 | East Greenland | Summer 2012 | Bourque et al. 2018 |
| Killer Whale | 48335 | East Greenland | Summer 2012 | Bourque et al. 2018 |
| Killer Whale | 48336 | East Greenland | Summer 2012 | Bourque et al. 2018 |
| Killer Whale | 48337 | East Greenland | Summer 2012 | Bourque et al. 2018 |
| Killer Whale | 48338 | East Greenland | Summer 2012 | Bourque et al. 2018 |
| Killer Whale | 48339 | East Greenland | Summer 2012 | Bourque et al. 2018 |
| Killer Whale | 48732 | East Greenland | Summer 2013 | Bourque et al. 2018 |
| Killer Whale | 48733 | East Greenland | Summer 2013 | Bourque et al. 2018 |
| Killer Whale | 48735 | East Greenland | Summer 2013 | Bourque et al. 2018 |
| Killer Whale | 48736 | East Greenland | Summer 2013 | Bourque et al. 2018 |
| Killer Whale | 51601 | East Greenland | Summer 2014 | Bourque et al. 2018 |
| Killer Whale | 51606 | East Greenland | Summer 2014 | Bourque et al. 2018 |
| Killer Whale | 51607 | East Greenland | Summer 2014 | Bourque et al. 2018 |
| Killer Whale | 51610 | East Greenland | Summer 2014 | Bourque et al. 2018 |
| Killer Whale | 51613 | East Greenland | Summer 2014 | Bourque et al. 2018 |
| Killer Whale | 40888 | Faroe Islands | Winter 2008 | Bourque et al. 2018 |
| Killer Whale | 40889 | Faroe Islands | Winter 2008 | Bourque et al. 2018 |
| Killer Whale | SW080429 | SeaWorld | 2008 | Bourque et al. 2018 |
| Killer Whale | SW100500 | SeaWorld | 2010 | Bourque et al. 2018 |
| Killer Whale | SW100743 | SeaWorld | 2010 | Bourque et al. 2018 |
| Killer Whale | SW100830 | SeaWorld | 2010 | Bourque et al. 2018 |
| Prey for the QFASA library on the managed-care whales | | | | |
| Capelin (n=10) | SWCapelin-1-10 | SeaWorld |  | This paper |
| Pacific Herring (n=10) | SWHerring-11-20 | SeaWorld |  | This paper |
| Mackerel (n=10) | SWMackerel-21-30 | SeaWorld |  | This paper |
| Sockeye Salmon (n=4) | SWSalmon-51-54 | SeaWorld |  | This paper |
| Prey for the QFASA library on the free-ranging killer whales | | | | |
| Atlantic Herring (n=10) | GLHerring-31-40 | East Greenland |  | This paper |
| Atlantic Mackerel (n=10) | GLMackerel-41-50 | East Greenland |  | This paper |
| Bearded Seal |  |  |  |  |
| n=8 | SGBS1-SGBS8 | South Greenland |  | McKinney et al. 2013 |
| n=19 | SIP107-SIP317 | Davis Strait |  | Thiemann et al. 2008 |
| n=28 | UMP197-UMP480 | Davis Strait |  | Thiemann et al. 2008 |
| Harp Seal |  |  |  |  |
| n=135 | SIP001-SIP283 | Davis Strait |  | Thiemann et al. 2008 |
| n=104 | Tucker001-Tucker 114 | Davis Strait |  | Thiemann et al. 2008 |
| Hooded Seal |  |  |  |  |
| n=17 | SIP098-SIP272 | Davis Strait |  | Thiemann et al. 2008 |
| n=15 | Tucker115-Tucker133 | Davis Strait |  | Thiemann et al. 2008 |
| Ringed Seal |  |  |  |  |
| n=50 | EGRS24931-EGRS34946 | East Greenland |  | Mckinney et al. 2013 |
| n=54 | SIP227-SIP319 | Davis Strait |  | Thiemann et al. 2008 |
| n=28 | UMP332-333 | Davis Strait |  | Thiemann et al. 2008 |
| Narwhal (n=16) | 53801-53846 | East Greenland |  | Unpublished |
| Bowhead Whale (n=62) | 01KK3-01SA1 | Alaska | 1997-2002 | Budge et al. 2008 |
| Minke Whale (n=5) | Ba_001-2017/0001 | East Greenland | 2000-2017 | Unpublished |

Fatty acid (FA) and fat content analyses

FAs were extracted and quantified as described in previous studies ^2,6^. In brief, for marine mammals, lipids were extracted from blubber using the Folch method ^7^. Whole fish were first homogenized in a food processor, extracted via a modified Folch and filtered (Budge et al 2006). All marine mammal and fish FA extracts were *trans*-esterified using the Hilditch reagent to produce FA methyl esters (FAMEs). The FAMEs were separated, identified, and the mass percentage of each of 69 FAs was quantified by gas chromatography on an Agilent 8860 system (Santa Clara, CA, USA) with flame ionization detection (GC-FID). Each FA is named using the nomenclature x:y*n*z, where x is the length of the carbon chain, y is the number of double bonds, and z is the position of the first double bond from the methyl (‘n’) end of the chain.

Relative fat contents of prey are required in the QFASA model to weight their relative contribution to FA signature. These were determined gravimetrically in fish during extraction^6^. For all marine mammal prey, given the lack of available data across species, we used a fat percentage of 30%, which was the average fat percentage calculated for the whole body of harbor seals.

Quality control of FA analyses, as previously described ^1^, included the extraction and analysis of a standard reference material, SRM1945 pilot whale blubber, from the US National Institute of Standards and Technology (NIST), with each batch of 11 samples. The SRM was run 16 times, and the relative standard deviation of the FA values averaged 16% compared to the published 27 individual FA values ^1,8^. All fish samples were extracted and analyzed in duplicate. The average FA values of duplicates were used, and the percent difference of the retained duplicates averaged 20%. A mixed standard containing 18 FAME (68B; Nu-Chek Prep, Elysian, MN, USA) was run for additional quality control for the fish samples; the average relative error was 5.6% (*n* = 5).

Table S2: Fatty acid (FA) percentages (mean ± SE) in the managed-care killer whales (n=4) and their prey items. Only FA greater than 0.1% are shown. The prey species were: Pacific herring (*Clupea pallasii)*, capelin *(Mallotus villosus)*, Pacific mackerel *(Scomber japonicus)*, and sockeye salmon *(Oncorhynchus nerka).* Italicized FAs are the extended dietary set and bold FAs are the dietary set. Normal font represents non-dietary FAs (which were not included in the QFASA analyses).

|  | **Capelin (n=10)** | **Pacific herring (n=10)** | **Pacific mackerel (n=10)** | **Sockeye salmon (n=4)** | **Killer whale (n=4)** |
| --- | --- | --- | --- | --- | --- |
| Saturated FA |  |  |  |  |  |
| *14:0* | 3.21 ± 0.29 | 4.75 ± 0.23 | 1.93 ± 0.12 | 2.08 ± 0.06 | 6.01 ± 0.24 |
| iso15:0 | 0.06 ± 0.01 | 0.14 ± 0.01 | 0.13 ± 0.01 | 0.12 ± 0.01 | 0.45 ± 0.05 |
| 15:0 | 0.20 ± 0.01 | 0.41 ± 0.02 | 0.63 ± 0.03 | 0.33 ± 0.02 | 0.69 ± 0.09 |
| *16:0* | 15.47 ± 0.99 | 19.02 ± 0.75 | 15.77 ± 0.17 | 12.08 ± 0.39 | 6.39 ± 0.36 |
| *17:0* | 0.09 ± 0.01 | 0.22 ± 0.01 | 0.74 ± 0.01 | 0.24 ± 0.01 | 0.14 ± 0.02 |
| *18:0* | 1.89 ± 0.18 | 2.59 ± 0.11 | 7.80 ± 0.19 | 3.59 ± 0.16 | 1.29 ± 0.15 |
| ∑SFA | 20.93 ± 0.12 | 27.13 ± 0.09 | 27.01 ± 0.03 | 18.44 ± 0.07 | 14.97 ± 0.06 |
| Mono-Unsaturated FA |  |  |  |  |  |
| 16:1n11 | 0.29 ± 0.02 | 0.44 ± 0.02 | 0.59 ± 0.02 | 0.34 ± 0.03 | 1.57 ± 0.20 |
| 16:1n9 | 0.17 ± 0.01 | 0.16 ± 0.01 | 0.35 ± 0.02 | 0.38 ± 0.04 | 1.58 ± 0.26 |
| *16:1n7* | 5.01 ± 0.36 | 6.84 ± 0.18 | 2.31 ± 0.12 | 3.27 ± 0.33 | 13.33 ± 0.35 |
| 7Me16:0 | 0.30 ± 0.01 | 0.23 ± 0.03 | 0.16 ± 0.01 | 0.30 ± 0.04 | 0.24 ± 0.01 |
| ***16:2n4*** | 0.33 ± 0.02 | 0.23 ± 0.03 | 0.75 ± 0.03 | 0.32 ± 0.06 | 0.28 ± 0.02 |
| ***16:3n6*** | 0.22 ± 0.02 | 0.77 ± 0.06 | 0.07 ± <0.01 | 0.10 ± 0.02 | 0.53 ± 0.03 |
| 17:1 | 0.06 ± <0.01 | 0.28 ± 0.01 | 0.28 ± 0.01 | 0.31 ± 0.04 | 0.40 ± 0.02 |
| ***16:4n3*** | 0.07 ± 0.01 | 0.11 ± <0.01 | 0.16 ± 0.01 | 0.19 ± 0.01 | 0.11 ± 0.01 |
| ***16:4n1*** | 0.21 ± 0.03 | 0.68 ± 0.08 | 0.05 ± <0.01 | 0.04 ± 0.01 | 0.20 ± 0.02 |
| 18:1n11 | 0.77 ± 0.02 | 0.29 ± 0.05 | 0.06 ± 0.01 | 1.34 ± 0.18 | 7.29 ± 0.60 |
| *18:1n9* | 5.13 ± 0.19 | 15.83 ± 1.66 | 6.51 ± 0.19 | 16.04 ± 0.83 | 17.74 ± 0.63 |
| *18:1n7* | 2.67 ± 0.12 | 4.67 ± 0.31 | 2.82 ± 0.04 | 2.92 ± 0.33 | 2.38 ± 0.14 |
| 18:1n5 | 0.57 ± 0.02 | 0.32 ± 0.06 | 0.13 ± 0.01 | 0.61 ± 0.05 | 0.36 ± 0.02 |
| ***20:1n11*** | 0.74 ± 0.07 | 1.86 ± 0.41 | 0.20 ± 0.05 | 4.79 ± 1.28 | 4.63 ± 0.43 |
| ***20:1n9*** | 9.85 ± 1.11 | 3.71 ± 0.63 | 0.57 ± 0.06 | 2.13 ± 0.38 | 6.37 ± 0.28 |
| ***20:1n7*** | 1.00 ± 0.13 | 0.45 ± 0.03 | 0.23 ± 0.03 | 0.31 ± 0.10 | 0.30 ± 0.02 |
| ***22:1n11*** | 9.84 ± 1.42 | 6.73 ± 1.17 | 0.20 ± 0.04 | 3.87 ± 1.45 | 5.46 ± 0.13 |
| ***22:1n9*** | 1.30 ± 0.17 | 0.44 ± 0.05 | 0.19 ± 0.03 | 0.43 ± 0.17 | 0.52 ± 0.02 |
| 24:1n9 | 0.75 ± 0.02 | 0.55 ± 0.07 | 0.50 ± 0.03 | 0.47 ± 0.14 | 0.18 ± 0.01 |
| ∑MUFA | 39.29 ± 0.12 | 44.58 ± 0.14 | 16.14 ± 0.01 | 38.17 ± 0.21 | 63.46 ± 0.10 |
| Poly-Unsaturated FA |  |  |  |  |  |
| *18:2n6* | 0.82 ± 0.02 | 0.89 ± 0.05 | 1.56 ± 0.07 | 1.46 ± 0.09 | 2.33 ± 0.89 |
| ***18:3n6*** | 0.01 ± <0.01 | 0.05 ± <0.01 | 0.28 ± 0.01 | 0.03 ± <0.01 | 0.10 ± 0.01 |
| ***18:3n3*** | 0.27 ± 0.02 | 0.51 ± 0.03 | 1.42 ± 0.09 | 0.88 ± 0.06 | 3.59 ± 0.72 |
| ***18:4n3*** | 0.55 ± 0.03 | 0.83 ± 0.05 | 1.95 ± 0.16 | 0.83 ± 0.07 | 0.54 ± 0.04 |
| ***18:4n1*** | 0.16 ± 0.01 | 0.14 ± 0.01 | 0.01 ± <0.01 | 0.11 ± 0.03 | 0.16 ± 0.02 |
| ***20:2n6*** | 0.21 ± 0.05 | 0.19 ± 0.04 | 0.60 ± 0.06 | 0.34 ± 0.03 | 0.13 ± 0.01 |
| ***20:4n6*** | 0.52 ± 0.03 | 0.95 ± 0.04 | 2.78 ± 0.13 | 1.28 ± 0.09 | 0.24 ± 0.04 |
| ***20:3n3*** | 0.09 ± 0.02 | 0.10 ± 0.01 | 0.28 ± 0.02 | 0.16 ± 0.01 | 0.10 ± 0.03 |
| ***20:4n3*** | 0.35 ± 0.01 | 0.34 ± 0.01 | 0.58 ± 0.02 | 1.49 ± 0.24 | 0.34 ± 0.04 |
| ***20:5n3*** | 13.34 ± 0.78 | 11.35 ± 0.29 | 9.65 ± 0.28 | 12.12 ± 1.87 | 2.25 ± 0.29 |
| ***21:5n3*** | 0.34 ± 0.02 | 0.35 ± 0.02 | 0.22 ± 0.01 | 0.26 ± 0.03 | 0.14 ± 0.02 |
| ***22:4n6*** | 0.03 ± 0.01 | 0.09 ± 0.01 | 0.40 ± 0.03 | 0.12 ± 0.01 | 0.12 ± 0.02 |
| *22:5n3* | 1.79 ± 0.13 | 1.03 ± 0.06 | 1.78 ± 0.06 | 4.14 ± 0.57 | 1.53 ± 0.30 |
| ***22:6n3*** | 19.83 ± 1.42 | 9.66 ± 0.41 | 32.73 ± 0.67 | 18.58 ± 0.67 | 3.87 ± 0.59 |
| ∑PUFA | 38.30 ± 0.13 | 26.47 ± 0.04 | 54.25 ± 0.06 | 41.80 ± 0.25 | 15.44 ± 0.15 |
| **∑21 Dietary** | 60.07 ± 5.39 | 40.41 ± 3.45 | 54.90 ± 1.81 | 49.84 ± 6.66 | 32.31 ± 3.68 |
| *∑30 Dietary extended* | 95.34 ± 1.64 | 95.36 ± 1.55 | 94.57 ± 2.08 | 94.22 ± 2.46 | 81.11 ± 2.06 |

Figure S1: Representation of the blubber layers in our killer whale samples, including full blubber, outer and inner blubber, as well as the typical depth of a biopsy (in dark grey)

.

Figure S2: Blubber depth variation in the FA CCs calculated from the managed-care killer whales and their known diet items for the dietary (with asterisk) and extended FAs greater than 0.1% of total FAs. Layer 1 is closest to muscle, while layer 10 is closest to skin.

Table S3: Calibration coefficients (CCs) used in the QFASA simulations. Killer whale CCs were calculated for the managed-care killer whales from this study. CCs shown here are for the dietary (bold) and extended dietary FAs that were greater than 0.1% total FAs. Layer 1 is closest to muscle, while layer 10 is closest to skin.

| Fatty Acid | Full blubber | Layer 1 | Inner blubber  (Layers 1– 4) | Outer blubber  (Layers 6–10) | Layer 10 |
| --- | --- | --- | --- | --- | --- |
| 14:0 | 1.516 | 1.535 | 1.654 | 1.339 | 1.300 |
| 16:0 | 0.377 | 0.460 | 0.423 | 0.310 | 0.319 |
| 16:1n7 | 2.261 | 1.210 | 1.607 | 3.034 | 3.104 |
| **16:2n4** | 1.034 | 0.843 | 0.904 | 1.219 | 1.264 |
| **16:3n6** | 1.067 | 0.908 | 0.976 | 1.176 | 1.234 |
| 17:0 | 0.807 | 1.423 | 1.032 | 0.476 | 0.499 |
| **16:4n3** | 1.177 | 1.025 | 1.115 | 1.279 | 1.171 |
| **16:4n1** | 0.463 | 0.595 | 0.542 | 0.336 | 0.267 |
| 18:0 | 0.551 | 0.898 | 0.706 | 0.366 | 0.420 |
| 18:1n9 | 1.646 | 1.293 | 1.484 | 1.844 | 1.875 |
| 18:1n7 | 0.644 | 0.685 | 0.677 | 0.601 | 0.628 |
| **18:2n6** | 2.623 | 2.025 | 2.418 | 2.722 | 2.682 |
| **18:3n6** | 3.180 | 4.355 | 3.742 | 2.038 | 1.516 |
| **18:3n3** | 8.398 | 2.113 | 3.411 | 13.831 | 10.771 |
| **18:4n3** | 0.752 | 0.965 | 0.907 | 0.490 | 0.406 |
| **18:4n1** | 1.143 | 1.330 | 1.280 | 0.810 | 0.729 |
| **20:1n11** | 3.571 | 4.860 | 4.428 | 2.895 | 3.416 |
| **20:1n9** | 1.111 | 1.713 | 1.502 | 0.692 | 0.747 |
| **20:1n7** | 0.481 | 0.873 | 0.702 | 0.246 | 0.265 |
| **20:2n6** | 0.716 | 0.993 | 0.886 | 0.481 | 0.477 |
| **20:4n6** | 0.307 | 0.323 | 0.308 | 0.292 | 0.349 |
| **20:3n3** | 1.034 | 0.670 | 0.841 | 1.091 | 0.667 |
| **20:4n3** | 0.871 | 1.003 | 1.000 | 0.609 | 0.574 |
| **20:5n3** | 0.187 | 0.200 | 0.201 | 0.137 | 0.125 |
| **22:1n11** | 0.753 | 1.465 | 1.106 | 0.423 | 0.507 |
| **22:1n9** | 0.718 | 1.445 | 1.107 | 0.354 | 0.393 |
| **21:5n3** | 0.399 | 0.620 | 0.545 | 0.180 | 0.130 |
| **22:4n6** | 1.841 | 1.940 | 1.590 | 1.954 | 1.787 |
| 22:5n3 | 1.073 | 2.140 | 1.614 | 0.444 | 0.450 |
| **22:6n3** | 0.280 | 0.468 | 0.408 | 0.118 | 0.118 |

Table S4: Leave-one-prey-out (LOPO) simulations on the prey library of the managed-care killer whales to establish a potential overlap of prey FA signatures. Numbers in bold represent the percentage of prey correctly identified.

| **Dietary FA (21) Aitchison distance** | | | | | **Dietary FA (21) KL distance** | | | | | |
| --- | --- | --- | --- | --- | --- | --- | --- | --- | --- | --- |
|  | Capelin | Herring | Mackerel | Salmon |  | Capelin | Herring | Mackerel | Salmon |  |
| Capelin | **94.63%** | 2.20% | 0.32% | 2.85% | Capelin | **83.85%** | 4.20% | 11.65% | 0.30% |  |
| Herring | 6.57% | **84.70%** | 3.73% | 5.01% | Herring | 11.10% | **82.88%** | 3.45% | 2.56% |  |
| Mackerel | 0.72% | 0.18% | **98.78%** | 0.33% | Mackerel | 0.52% | 0.06% | **98.90%** | 0.51% |  |
| Salmon | 6.30% | 2.22% | 4.63% | **86.85%** | Salmon | 4.95% | 8.15% | 14.78% | **72.12%** |  |
|  |  |  |  |  |  |  |  |  |  |  |
| **Dietary Extended FA (30) Aitchison Distance** | | | | | **Dietary Extended FA (30) KL Distance** | | | | | |
|  | Capelin | Herring | Mackerel | Salmon |  | Capelin | Herring | Mackerel | Salmon |  |
| Capelin | **94.12%** | 2.23% | 0.23% | 3.42% | Capelin | **85.88%** | 3.03% | 10.08% | 1.01% |  |
| Herring | 7.18% | **86.36%** | 3.23% | 3.22% | Herring | 13.25% | **82.32%** | 2.05% | 2.38% |  |
| Mackerel | 0.71% | 0.19% | **98.78%** | 0.32% | Mackerel | 0.46% | 0.15% | **98.95%** | 0.43% |  |
| Salmon | 5.90% | 2.01% | 4.31% | **87.78%** | Salmon | 3.75% | 5.38% | 9.27% | **81.60%** |  |

Table S5: QFASA diet estimates for managed-care killer whales (n=4) using the full blubber FA signatures and no CCs. The actual diet of the managed care killer whales consisted of 32% capelin, 60% herring, 4% mackerel and 4% salmon.

| **Full Blubber - No CCs - Dietary FA (21) - KL distance** | | | | | | | |
| --- | --- | --- | --- | --- | --- | --- | --- |
|  | Killer whale - SW080429 | Killer whale - SW100500 | Killer whale - SW100743 | Killer whale - SW100830 | Mean ± SE | % Error | Total % Error |
| Capelin | 0.00 | 0.00 | 0.00 | 0.00 | <0.01 ± 0.29 | 100.00 | 91.67 |
| Herring | 100.00 | 100.00 | 100.00 | 100.00 | 100 ± 4.11 | 66.67 |  |
| Mackerel | 0.00 | 0.00 | 0.00 | 0.00 | <0.01 ± <0.01 | 100.00 |  |
| Salmon | 0.00 | 0.00 | 0.00 | 0.00 | <0.01 ± 4.10 | 100.00 |  |
| **Full Blubber - No CCs - Dietary FA (21) - Aitchison distance** | | | | | | | |
|  | Killer whale - SW080429 | Killer whale - SW100500 | Killer whale - SW100743 | Killer whale - SW100830 | Mean ± SE | % Error | Total % Error |
| Capelin | 7.41 | 7.14 | 10.00 | 6.35 | 7.73 ± 4.85 | 75.86 | 182.50 |
| Herring | 52.90 | 69.82 | 49.19 | 61.00 | 58.23 ± 11.45 | 2.96 |  |
| Mackerel | 11.53 | 4.87 | 7.00 | 10.80 | 8.55 ± 2.18 | 113.74 |  |
| Salmon | 28.16 | 18.17 | 33.81 | 21.85 | 25.50 ± 9.72 | 537.46 |  |

Table S6: QFASA diet estimates for managed-care killer whales (n=4) using marine-fed mink and grey seal CC. Simulations resulted in inaccurate estimates compared to the killer whales’ actual diet which consisted of 32% capelin, 60% herring, 4% mackerel and 4% salmon. The mean and SE were bootstrapped 100 times.

| **Full Blubber - Marine-fed Mink CCs - Dietary FA (21) - KL distance** | | | | | | | |
| --- | --- | --- | --- | --- | --- | --- | --- |
|  | Killer whale - SW080429 | Killer whale - SW100500 | Killer whale - SW100743 | Killer whale - SW100830 | Mean ± SE | % Error | Total % Error |
| Capelin | 4.30 | 15.79 | 19.25 | 1.05 | 10.10 ± 5.81 | 68.44 | 79.35 |
| Herring | 95.70 | 84.21 | 80.62 | 98.95 | 89.87 ± 6.12 | 49.78 |  |
| Mackerel | 0.00 | 0.00 | 0.13 | 0.00 | 0.03 ± 1.28 | 99.18 |  |
| Salmon | 0.00 | 0.00 | 0.00 | 0.00 | <0.01 ± 0.41 | 100.00 |  |
| **Full Blubber - Marine-fed Mink CCs - Dietary FA (21) - Aitchison distance** | | | | | | | |
|  | Killer whale - SW080429 | Killer whale - SW100500 | Killer whale - SW100743 | Killer whale - SW100830 | Mean ± SE | % Error | Total % Error |
| Capelin | 4.41 | 2.52 | 5.69 | 2.72 | 3.83 ± 4.65 | 88.02 | 113.70 |
| Herring | 68.85 | 85.83 | 63.83 | 79.32 | 74.46 ± 8.32 | 24.10 |  |
| Mackerel | 14.29 | 4.78 | 7.16 | 13.12 | 9.83 ± 2.77 | 145.95 |  |
| Salmon | 12.46 | 6.87 | 23.32 | 4.83 | 11.87 ± 5.28 | 196.75 |  |
| **Full Blubber - Gray seal CCs - Dietary FA (21) - KL distance** | | | | | | | |
|  | Killer whale - SW080429 | Killer whale - SW100500 | Killer whale - SW100743 | Killer whale - SW100830 | Mean ± SE | % Error | Total % Error |
| Capelin | 0.09 | 12.69 | 18.87 | 0.00 | 7.91 ± 6.72 | 75.27 | 82.19 |
| Herring | 99.91 | 87.31 | 81.13 | 100.00 | 92.09 ± 7.30 | 53.48 |  |
| Mackerel | 0.00 | 0.00 | 0.00 | 0.00 | <0.01 ± <0.01 | 100.00 |  |
| Salmon | 0.00 | 0.00 | 0.00 | 0.00 | <0.01 ± 1.30 | 100.00 |  |
| **Full Blubber - Gray seal CCs - Dietary FA (21) - Aitchison distance** | | | | | | | |
|  | Killer whale - SW080429 | Killer whale - SW100500 | Killer whale - SW100743 | Killer whale - SW100830 | Mean ± SE | % Error | Total % Error |
| Capelin | 31.38 | 30.23 | 33.26 | 26.97 | 30.46 ± 6.93 | 4.81 | 47.72 |
| Herring | 58.20 | 66.08 | 60.42 | 64.28 | 62.25 ± 8.70 | 3.75 |  |
| Mackerel | 10.42 | 3.69 | 6.32 | 8.75 | 7.29 ± 2.38 | 82.31 |  |
| Salmon | 0.00 | 0.00 | 0.00 | 0.00 | <0.01 ± 1.36 | 100.00 |  |
| **Inner Blubber - Marine-fed Mink CCs - Dietary FA (21) - KL distance** | | | | | | | |
|  | Killer whale - SW080429 | Killer whale - SW100500 | Killer whale - SW100743 | Killer whale - SW100830 | Mean ± SE | % Error | Total % Error |
| Capelin | 29.80 | 40.63 | 35.87 | 38.32 | 36.16 | 12.99 | 54.85 |
| Herring | 70.20 | 59.37 | 64.13 | 61.68 | 63.84 | 6.41 |  |
| Mackerel | 0.00 | 0.00 | 0.00 | 0.00 | <0.01 ± 0.17 | 100.00 |  |
| Salmon | 0.00 | 0.00 | 0.00 | 0.00 | <0.01 ± 1.04 | 100.00 |  |
| **Inner Blubber - Marine-fed Mink CCs - Dietary FA (21) - Aitchison distance** | | | | | | | |
|  | Killer whale - SW080429 | Killer whale - SW100500 | Killer whale - SW100743 | Killer whale - SW100830 | Mean ± SE | % Error | Total % Error |
| Capelin | 25.44 | 15.53 | 13.51 | 23.40 | 19.47 ± 6.60 | 39.15 | 65.83 |
| Herring | 50.73 | 76.50 | 60.86 | 67.18 | 63.82 ± 8.20 | 6.36 |  |
| Mackerel | 12.77 | 4.89 | 6.42 | 6.42 | 7.62 ± 2.04 | 90.61 |  |
| Salmon | 11.06 | 3.08 | 19.21 | 3.01 | 9.09 ± 3.83 | 127.21 |  |
| **Inner Blubber - Marine-fed Mink CCs - Dietary FA (21) - KL distance** | | | | | | | |
|  | Killer whale - SW080429 | Killer whale - SW100500 | Killer whale - SW100743 | Killer whale - SW100830 | Mean ± SE | % Error | Total % Error |
| Capelin | 29.80 | 40.63 | 35.87 | 38.32 | 36.16 | 12.99 | 54.85 |
| Herring | 70.20 | 59.37 | 64.13 | 61.68 | 63.84 | 6.41 |  |
| Mackerel | 0.00 | 0.00 | 0.00 | 0.00 | <0.01 ± 0.17 | 100.00 |  |
| Salmon | 0.00 | 0.00 | 0.00 | 0.00 | <0.01 ± 1.04 | 100.00 |  |
| **Inner Blubber - Marine-fed Mink CCs - Dietary FA (21) - Aitchison distance** | | | | | | | |
|  | Killer whale - SW080429 | Killer whale - SW100500 | Killer whale - SW100743 | Killer whale - SW100830 | Mean ± SE | % Error | Total % Error |
| Capelin | 25.44 | 15.53 | 13.51 | 23.40 | 19.47 ± 6.60 | 39.15 | 65.83 |
| Herring | 50.73 | 76.50 | 60.86 | 67.18 | 63.82 ± 8.20 | 6.36 |  |
| Mackerel | 12.77 | 4.89 | 6.42 | 6.42 | 7.62 ± 2.04 | 90.61 |  |
| Salmon | 11.06 | 3.08 | 19.21 | 3.01 | 9.09 ± 3.83 | 127.21 |  |
| **Inner Blubber - Gray seal CCs - Dietary FA (21) - KL distance** | | | | | | | |
|  | Killer whale - SW080429 | Killer whale - SW100500 | Killer whale - SW100743 | Killer whale - SW100830 | Mean ± SE | % Error | Total % Error |
| Capelin | 25.33 | 39.49 | 37.73 | 33.58 | 34.03 ± 10.38 | 6.35 | 53.29 |
| Herring | 74.67 | 60.51 | 61.80 | 66.42 | 65.85 ± 11.98 | 9.75 |  |
| Mackerel | 0.00 | 0.00 | 0.00 | 0.00 | <0.01 ± <0.01 | 100.00 |  |
| Salmon | 0.00 | 0.00 | 0.47 | 0.00 | <0.01 ± 3.62 | 97.06 |  |
| **Inner Blubber - Gray seal CCs - Dietary FA (21) - Aitchison distance** | | | | | | | |
|  | Killer whale - SW080429 | Killer whale - SW100500 | Killer whale - SW100743 | Killer whale - SW100830 | Mean ± SE | % Error | Total % Error |
| Capelin | 47.48 | 41.22 | 40.43 | 44.08 | 43.30 ± 7.13 | 35.33 | 47.07 |
| Herring | 44.03 | 54.95 | 54.16 | 51.52 | 51.17 ± 8.87 | 14.72 |  |
| Mackerel | 8.49 | 3.83 | 5.41 | 4.39 | 5.53 ± 2.00 | 38.24 |  |
| Salmon | 0.00 | 0.00 | 0.00 | 0.00 | <0.01 ± <0.01 | 100.00 |  |
| **Outer Blubber - Marine-fed Mink CCs - Dietary FA (21) - KL distance** | | | | | | | |
|  | Killer whale - SW080429 | Killer whale - SW100500 | Killer whale - SW100743 | Killer whale - SW100830 | Mean ± SE | % Error | Total % Error |
| Capelin | 0.00 | 0.00 | 0.00 | 0.00 | <0.01 ± 0.34 | 100.00 | 91.67 |
| Herring | 100.00 | 100.00 | 100.00 | 100.00 | 100 ± 1.42 | 66.67 |  |
| Mackerel | 0.00 | 0.00 | 0.00 | 0.00 | <0.01 ± 1.29 | 100.00 |  |
| Salmon | 0.00 | 0.00 | 0.00 | 0.00 | <0.01 ± <0.01 | 100.00 |  |
| **Outer Blubber - Marine-fed Mink CCs - Dietary FA (21) - Aitchison distance** | | | | | | | |
|  | Killer whale - SW080429 | Killer whale - SW100500 | Killer whale - SW100743 | Killer whale - SW100830 | Mean ± SE | % Error | Total % Error |
| Capelin | 0.00 | 0.00 | 0.00 | 0.00 | <0.01 ± 0.42 | 100.00 | 157.47 |
| Herring | 70.12 | 78.68 | 63.62 | 73.89 | 71.58 ± 5.95 | 19.30 |  |
| Mackerel | 17.78 | 7.38 | 10.25 | 22.28 | 14.42 ± 3.64 | 260.49 |  |
| Salmon | 12.11 | 13.94 | 26.13 | 3.83 | 14.00 ± 7.07 | 250.08 |  |
| **Outer Blubber - Gray seal CCs - Dietary FA (21) - KL distance** | | | | | | | |
|  | Killer whale - SW080429 | Killer whale - SW100500 | Killer whale - SW100743 | Killer whale - SW100830 | Mean ± SE | % Error | Total % Error |
| Capelin | 0.00 | 0.00 | 0.00 | 0.00 | <0.01 ± 0.45 | 100.00 | 91.67 |
| Herring | 100.00 | 100.00 | 100.00 | 100.00 | 100 ± 0.45 | 66.67 |  |
| Mackerel | 0.00 | 0.00 | 0.00 | 0.00 | <0.01 ± <0.01 | 100.00 |  |
| Salmon | 0.00 | 0.00 | 0.00 | 0.00 | <0.01 ± 0.03 | 100.00 |  |
| **Outer Blubber - Gray seal CCs - Dietary FA (21) - Aitchison distance** | | | | | | | |
|  | Killer whale - SW080429 | Killer whale - SW100500 | Killer whale - SW100743 | Killer whale - SW100830 | Mean ± SE | % Error | Total % Error |
| Capelin | 18.09 | 11.23 | 14.53 | 10.10 | 13.49 ± 6.954 | 57.85 | 70.45 |
| Herring | 70.73 | 86.65 | 78.98 | 78.86 | 78.81 ± 9.02 | 31.35 |  |
| Mackerel | 11.18 | 2.12 | 6.48 | 11.04 | 7.70 ± 2.84 | 92.60 |  |
| Salmon | 0.00 | 0.00 | 0.00 | 0.00 | <0.01 ± 0.95 | 100.00 |  |

Table S7: Two-by-two comparison of the diet estimates (in %) using the FAs from two managed-care killer whales and the CCs from the other two remaining killer whales (n=4 total) on the inner and outer blubber FA signatures and the inner and outer blubber CCs.

| **Full Blubber - Full CCs - Dietary (21) FAs - KL Distance** | | | | | | | | | | |
| --- | --- | --- | --- | --- | --- | --- | --- | --- | --- | --- |
| Fatty acids used | CC used | Capelin mean | Capelin SE | Herring mean | Herring SE | Mackerel mean | Mackerel SE | Salmon mean | Salmon SE |  |
| SW080429 + SW100830 | SW100500 + SW100743 | 24.99 | 5.87 | 72.15 | 6.37 | 2.85 | 2.78 | 0.00 | 1.21 |  |
| SW100500 + SW100743 | SW080429 + SW100830 | 52.60 | 5.47 | 38.35 | 8.97 | 0.00 | 0.19 | 9.05 | 5.19 |  |
| SW080429 + SW100500 | SW100830 + SW100743 | 34.22 | 6.86 | 65.70 | 6.94 | 0.07 | 2.07 | 0.00 | 0.35 |  |
| SW100830 + SW100743 | SW080429 + SW100500 | 33.23 | 9.95 | 53.16 | 13.24 | 3.84 | 2.80 | 9.78 | 6.28 |  |
| SW080429 + SW100743 | SW100830 + SW100500 | 37.25 | 8.25 | 56.48 | 10.68 | 0.55 | 1.80 | 5.73 | 4.36 |  |
| SW100830 + SW100500 | SW080429 + SW100743 | 29.00 | 7.56 | 68.24 | 6.89 | 2.76 | 2.61 | 0.00 | 2.17 |  |
| Actual diet |  | 32.00 | – | 60.00 | – | 4.00 | – | 4.00 | – |  |
| Average simulations |  | 35.21 | 7.33 | 59.01 | 8.85 | 1.68 | 2.04 | 4.09 | 3.26 |  |
| %Error |  | 10.04 |  | 1.64 |  | 58.03 |  | 2.31 |  |  |
| Total % Error |  | 18.01 |  |  |  |  |  |  |  |  |
|  |  |  |  |  |  |  |  |  |  |  |
| **Full Blubber - Full CCs - Dietary (21) FAs - Aitchison Distance** | | | | | | | | | | |
| Fatty acids used | CC used | Capelin mean | Capelin SE | Herring mean | Herring SE | Mackerel mean | Mackerel SE | Salmon mean | Salmon SE |  |
| SW080429 + SW100830 | SW100500 + SW100743 | 37.36 | 3.21 | 60.29 | 3.62 | 0.00 | 0.18 | 2.35 | 2.36 |  |
| SW100500 + SW100743 | SW080429 + SW100830 | 34.55 | 3.66 | 58.48 | 5.57 | 2.90 | 1.26 | 4.07 | 2.65 |  |
| SW080429 + SW100500 | SW100830 + SW100743 | 33.67 | 4.16 | 57.65 | 6.28 | 1.75 | 1.34 | 6.94 | 4.09 |  |
| SW100830 + SW100743 | SW080429 + SW100500 | 38.77 | 3.37 | 58.30 | 4.13 | 1.27 | 1.17 | 1.66 | 2.20 |  |
| SW080429 + SW100743 | SW100830 + SW100500 | 32.76 | 3.26 | 66.05 | 2.73 | 1.19 | 0.91 | 0.00 | 0.53 |  |
| SW100830 + SW100500 | SW080429 + SW100743 | 35.94 | 3.44 | 59.62 | 4.13 | 1.28 | 1.13 | 3.16 | 2.27 |  |
| Actual diet |  | 32.00 | – | 60.00 | – | 4.00 | – | 4.00 | – |  |
| Average simulations |  | 35.51 | 3.52 | 60.06 | 4.41 | 1.40 | 1.00 | 3.03 | 2.35 |  |
| % Error |  | 10.96 |  | 0.11 |  | 65.04 |  | 24.24 |  |  |
| Total % Error |  | 25.09 |  |  |  |  |  |  |  |  |
|  |  |  |  |  |  |  |  |  |  |  |
| **Inner Blubber - Inner CCs - Dietary (21) FAs - KL Distance** | | | | | | | | | | |
| Fatty acids used | CC used | Capelin mean | Capelin SE | Herring mean | Herring SE | Mackerel mean | Mackerel SE | Salmon mean | Salmon SE |  |
| SW080429 + SW100830 | SW100500 + SW100743 | 32.95 | 5.54 | 65.97 | 5.60 | 1.08 | 2.17 | 0.00 | 1.54 |  |
| SW100500 + SW100743 | SW080429 + SW100830 | 38.41 | 5.72 | 49.05 | 5.95 | 5.56 | 3.40 | 6.98 | 5.49 |  |
| SW080429 + SW100500 | SW100830 + SW100743 | 33.44 | 7.00 | 62.75 | 6.47 | 3.82 | 3.26 | 0.00 | 0.75 |  |
| SW100830 + SW100743 | SW080429 + SW100500 | 39.67 | 5.71 | 46.38 | 7.94 | 2.58 | 2.42 | 11.37 | 5.40 |  |
| SW080429 + SW100743 | SW100830 + SW100500 | 27.56 | 5.52 | 58.20 | 6.77 | 10.13 | 4.07 | 4.11 | 4.39 |  |
| SW100830 + SW100500 | SW080429 + SW100743 | 44.18 | 4.55 | 54.93 | 6.29 | 0.00 | 0.78 | 0.89 | 2.97 |  |
| Actual diet |  | 32.00 | – | 60.00 | – | 4.00 | – | 4.00 | – |  |
| Average simulations |  | 36.04 | 5.67 | 56.21 | 6.50 | 3.86 | 2.68 | 3.89 | 3.42 |  |
| %Error |  | 12.61 |  | 6.31 |  | 3.45 |  | 2.71 |  |  |
| Total % Error |  | 6.27 |  |  |  |  |  |  |  |  |
|  |  |  |  |  |  |  |  |  |  |  |
| **Inner Blubber - Inner CCs - Dietary (21) FAs - Aitchison Distance** | | | | | | | | | | |
| Fatty acids used | CC used | Capelin mean | Capelin SE | Herring mean | Herring SE | Mackerel mean | Mackerel SE | Salmon mean | Salmon SE |  |
| SW080429 + SW100830 | SW100500 + SW100743 | 43.77 | 2.79 | 50.49 | 5.87 | 2.35 | 1.43 | 3.38 | 3.20 |  |
| SW100500 + SW100743 | SW080429 + SW100830 | 31.93 | 5.27 | 62.04 | 4.70 | 0.00 | 0.15 | 6.03 | 5.17 |  |
| SW080429 + SW100500 | SW100830 + SW100743 | 39.54 | 3.93 | 55.80 | 6.43 | 2.03 | 1.65 | 2.63 | 2.51 |  |
| SW100830 + SW100743 | SW080429 + SW100500 | 35.70 | 3.64 | 57.25 | 4.27 | 0.00 | 0.36 | 7.04 | 4.03 |  |
| SW080429 + SW100743 | SW100830 + SW100500 | 34.99 | 4.46 | 46.30 | 5.27 | 2.21 | 1.41 | 16.50 | 3.43 |  |
| SW100830 + SW100500 | SW080429 + SW100743 | 38.55 | 2.49 | 61.45 | 2.55 | 0.00 | 0.13 | 0.00 | 0.37 |  |
| Actual diet |  | 32.00 | – | 60.00 | – | 4.00 | – | 4.00 | – |  |
| Average simulations |  | 36.14 | 3.96 | 56.57 | 4.65 | 0.85 | 0.74 | 6.44 | 3.10 |  |
| %Error |  | 12.95 |  | 5.72 |  | 78.82 |  | 61.01 |  |  |
| Total % Error |  | 39.62 |  |  |  |  |  |  |  |  |
|  |  |  |  |  |  |  |  |  |  |  |
| **Outer Blubber - Outer CCs - Dietary (21) FAs - KL Distance** | | | | | | | | | | |
| Fatty acids used | CC used | Capelin mean | Capelin SE | Herring mean | Herring SE | Mackerel mean | Mackerel SE | Salmon mean | Salmon SE |  |
| SW080429 + SW100830 | SW100500 + SW100743 | 40.05 | 10.10 | 51.28 | 6.48 | 8.67 | 5.13 | 0.00 | 1.01 |  |
| SW100500 + SW100743 | SW080429 + SW100830 | 20.73 | 3.80 | 78.02 | 4.78 | 0.00 | 0.00 | 1.25 | 3.19 |  |
| SW080429 + SW100500 | SW100830 + SW100743 | 59.18 | 9.17 | 40.82 | 9.18 | 0.00 | 0.00 | 0.00 | 0.10 |  |
| SW100830 + SW100743 | SW080429 + SW100500 | 23.11 | 6.22 | 60.47 | 6.68 | 11.15 | 5.63 | 5.27 | 5.79 |  |
| SW080429 + SW100743 | SW100830 + SW100500 | 57.71 | 8.37 | 37.83 | 8.05 | 0.00 | 0.00 | 4.46 | 3.82 |  |
| SW100830 + SW100500 | SW080429 + SW100743 | 22.95 | 6.33 | 69.02 | 6.75 | 8.03 | 4.97 | 0.00 | 1.46 |  |
| Actual diet |  | 32.00 | – | 60.00 | – | 4.00 | – | 4.00 | – |  |
| Average simulations |  | 37.29 | 7.33 | 56.24 | 6.99 | 4.64 | 2.62 | 1.83 | 2.56 |  |
| %Error |  | 16.53 |  | 6.27 |  | 16.03 |  | 54.25 |  |  |
| Total % Error |  | 23.27 |  |  |  |  |  |  |  |  |
|  |  |  |  |  |  |  |  |  |  |  |
| **Outer Blubber - Outer CCs - Dietary (21) FAs - Aitchison Distance** | | | | | | | | | | |
| Fatty acids used | CC used | Capelin mean | Capelin SE | Herring mean | Herring SE | Mackerel mean | Mackerel SE | Salmon mean | Salmon SE |  |
| SW080429 + SW100830 | SW100500 + SW100743 | 37.39 | 7.19 | 54.12 | 7.78 | 8.49 | 1.45 | 0.00 | 1.23 |  |
| SW100500 + SW100743 | SW080429 + SW100830 | 54.53 | 4.39 | 45.47 | 4.71 | 0.00 | 0.00 | 0.00 | 2.06 |  |
| SW080429 + SW100500 | SW100830 + SW100743 | 50.57 | 3.72 | 47.83 | 4.63 | 1.60 | 1.18 | 0.00 | 1.29 |  |
| SW100830 + SW100743 | SW080429 + SW100500 | 29.77 | 3.40 | 60.93 | 5.02 | 3.90 | 2.14 | 5.40 | 3.99 |  |
| SW080429 + SW100743 | SW100830 + SW100500 | 48.27 | 4.41 | 43.62 | 4.15 | 3.01 | 1.91 | 5.09 | 3.92 |  |
| SW100830 + SW100500 | SW080429 + SW100743 | 32.42 | 5.03 | 65.64 | 3.79 | 1.94 | 1.53 | 0.00 | 0.68 |  |
| Actual diet |  | 32.00 | – | 60.00 | – | 4.00 | – | 4.00 | – |  |
| Average simulations |  | 42.16 | 4.69 | 52.94 | 5.01 | 3.16 | 1.37 | 1.75 | 2.19 |  |
| %Error |  | 31.74 |  | 11.77 |  | 21.05 |  | 56.26 |  |  |
| Total % Error |  | 30.21 |  |  |  |  |  |  |  |  |

Table S8: QFASA diet estimates for managed-care killer whales (n=4) layer-specific CCs derived from the same managed-care killer whales (average of the four individual CCs) with the Aitchison distance. Diet estimates were more consistent and accurate with the Aitchison distance compared to the Kullback-Leibler (KL) distance. The managed-care killer whales’ actual diet consisted of 32% capelin, 60% herring, 4% mackerel and 4% salmon. The mean and SE were bootstrapped 100 times. Layer 1 is closest to muscle, while layer 10 is closest to skin.

| **Full Blubber - Full CCs - Dietary FA (21) - Aitchison distance** | | | | | | | |
| --- | --- | --- | --- | --- | --- | --- | --- |
|  | Killer whale - SW080429 | Killer whale - SW100500 | Killer whale - SW100743 | Killer whale - SW100830 | Mean ± SE | % Error | Total % Error |
| Capelin | 35.86 | 39.94 | 40.01 | 30.72 | 36.63 ± 3.58 | 14.48 | 22.07 |
| Herring | 55.81 | 60.06 | 51.32 | 65.61 | 58.20 ± 4.10 | 3.00 |  |
| Mackerel | 2.85 | 0.00 | 0.00 | 2.54 | 1.35 ± 0.87 | 66.32 |  |
| Salmon | 5.48 | 0.00 | 8.67 | 1.13 | 3.58 ± 2.45 | 4.47 |  |
| **Layer 1 blubber - Layer 1 blubber CCs - Dietary FA (21) - Aitchison distance** | | | | | | | |
|  | Killer whale - SW080429 | Killer whale - SW100500 | Killer whale - SW100743 | Killer whale - SW100830 | Mean ± SE | % Error | Total % Error |
| Capelin | 47.62 | 35.21 | 30.23 | 45.57 | 39.66 ± 3.89 | 23.93 | 37.67 |
| Herring | 41.52 | 64.79 | 56.14 | 52.16 | 53.65 ± 4.69 | 10.58 |  |
| Mackerel | 1.70 | 0.00 | 2.39 | 0.00 | 1.01 ± 0.69 | 74.46 |  |
| Salmon | 9.16 | 0.00 | 11.25 | 2.27 | 5.67 ± 2.73 | 41.72 |  |
| **Layer 1 blubber - Inner blubber CCs - Dietary FA (21) - Aitchison distance** | | | | | | | |
|  | Killer whale - SW080429 | Killer whale - SW100500 | Killer whale - SW100743 | Killer whale - SW100830 | Mean ± SE | % Error | Total % Error |
| Capelin | 54.79 | 41.54 | 39.50 | 50.46 | 46.57 ± 3.31 | 45.54 | 41.94 |
| Herring | 38.70 | 58.28 | 51.99 | 49.54 | 49.63 ± 4.05 | 17.29 |  |
| Mackerel | 2.53 | 0.19 | 3.28 | 0.00 | 1.50 ± 0.83 | 62.55 |  |
| Salmon | 3.99 | 0.00 | 5.23 | 0.00 | 2.30 ± 1.89 | 42.39 |  |
| **Inner blubber - Inner blubber CCs - Dietary FA (21) - Aitchison distance** | | | | | | | |
|  | Killer whale - SW080429 | Killer whale - SW100500 | Killer whale - SW100743 | Killer whale - SW100830 | Mean ± SE | % Error | Total % Error |
| Capelin | 41.19 | 36.49 | 32.32 | 39.99 | 37.50 ± 2.88 | 17.18 | 29.83 |
| Herring | 48.60 | 63.51 | 55.35 | 59.71 | 56.79 ± 3.91 | 5.35 |  |
| Mackerel | 3.14 | 0.00 | 0.23 | 0.30 | 0.92 ± 0.88 | 77.01 |  |
| Salmon | 7.07 | 0.00 | 12.10 | 0.00 | 4.79 ± 2.96 | 19.79 |  |
| **Inner blubber - layer 1 CCs - Dietary FA (21) - Aitchison distance** | | | | | | | |
|  | Killer whale - SW080429 | Killer whale - SW100500 | Killer whale - SW100743 | Killer whale - SW100830 | Mean ± SE | % Error | Total % Error |
| Capelin | 32.15 | 29.14 | 23.76 | 33.61 | 29.66 ± 3.30 | 7.30 | 61.17 |
| Herring | 51.89 | 67.70 | 57.02 | 62.66 | 59.82 ± 4.47 | 0.30 |  |
| Mackerel | 2.07 | 0.00 | 0.00 | 0.00 | 0.52 ± 0.73 | 87.07 |  |
| Salmon | 13.89 | 3.16 | 19.22 | 3.73 | 10.00 ± 4.37 | 149.99 |  |
| **Outer layer blubber - Full CCs - Dietary FA (21) - Aitchison distance** | | | | | | | |
|  | Killer whale - SW080429 | Killer whale - SW100500 | Killer whale - SW100743 | Killer whale - SW100830 | Mean ± SE | % Error | Total % Error |
| Capelin | 17.83 | 17.20 | 16.43 | 8.23 | 14.92 ± 3.35 | 53.36 | 58.07 |
| Herring | 69.69 | 77.43 | 66.98 | 82.26 | 74.09 ± 4.79 | 23.48 |  |
| Mackerel | 3.84 | 0.00 | 0.00 | 5.70 | 2.38 ± 1.52 | 40.40 |  |
| Salmon | 8.64 | 5.36 | 16.59 | 3.81 | 8.60 ± 3.65 | 115.03 |  |
| **Outer layer blubber - Outer layer CCs - Dietary FA (21) - Aitchison distance** | | | | | | | |
|  | Killer whale - SW080429 | Killer whale - SW100500 | Killer whale - SW100743 | Killer whale - SW100830 | Mean ± SE | % Error | Total % Error |
| Capelin | 46.75 | 41.80 | 40.05 | 31.79 | 40.10 ± 3.90 | 25.30 | 32.13 |
| Herring | 48.45 | 58.20 | 52.74 | 63.61 | 55.75 ± 4.01 | 7.08 |  |
| Mackerel | 4.07 | 0.00 | 0.00 | 4.60 | 2.17 ± 1.20 | 45.77 |  |
| Salmon | 0.73 | 0.00 | 7.21 | 0.00 | 1.98 ± 1.96 | 50.39 |  |
| **Outer layer blubber - layer 10 CCs - Dietary FA (21) - Aitchison distance** | | | | | | | |
|  | Killer whale - SW080429 | Killer whale - SW100500 | Killer whale - SW100743 | Killer whale - SW100830 | Mean ± SE | % Error | Total % Error |
| Capelin | 41.03 | 34.09 | 34.62 | 26.07 | 33.95 ± 4.30 | 6.10 | 17.89 |
| Herring | 52.27 | 65.91 | 57.84 | 66.48 | 60.62 ± 4.45 | 1.04 |  |
| Mackerel | 6.70 | 0.00 | 1.83 | 7.45 | 4.00 ± 1.80 | 0.12 |  |
| Salmon | 0.00 | 0.00 | 5.71 | 0.00 | 1.43 ± 1.78 | 64.30 |  |
| **Layer 10 blubber - layer 10 CCs - Dietary FA (21) - Aitchison distance** | | | | | | | |
|  | Killer whale - SW080429-10 | Killer whale - SW100500-10 | Killer whale - SW100743-10 | Killer whale - SW100830-10 | Mean ± SE | % Error | Total % Error |
| Capelin | 41.43 | 55.69 | 38.62 | 21.75 | 39.37 ± 6.46 | 23.04 | 23.55 |
| Herring | 57.26 | 42.59 | 50.42 | 66.65 | 54.23 ± 5.53 | 9.61 |  |
| Mackerel | 1.31 | 0.00 | 6.56 | 0.00 | 1.97 ± 1.41 | 50.85 |  |
| Salmon | 0.00 | 1.72 | 4.40 | 11.60 | 4.43 ± 2.64 | 10.72 |  |
| **Layer 10 blubber - Outer layer CCs - Dietary FA (21) - Aitchison distance** | | | | | | | |
|  | Killer whale - SW080429-10 | Killer whale - SW100500-10 | Killer whale - SW100743-10 | Killer whale - SW100830-10 | Mean ± SE | % Error | Total % Error |
| Capelin | 46.57 | 63.34 | 43.71 | 29.49 | 45.78 ± 6.39 | 43.06 | 46.43 |
| Herring | 52.83 | 34.14 | 45.13 | 57.45 | 47.39 ± 5.89 | 21.02 |  |
| Mackerel | 0.00 | 0.00 | 3.94 | 0.00 | 0.98 ± 0.84 | 75.40 |  |
| Salmon | 0.59 | 2.52 | 7.22 | 13.06 | 5.85 ± 3.03 | 46.25 |  |

Table S9: QFASA diet estimates for managed-care killer whales (n=4) layer-specific CCs derived from the same managed-care killer whales (average of the four individual CCs) with the KL distance. Diet estimates were more consistent and accurate with the Aitchison distance compared to the KL distance. The managed-care killer whales’ actual diet consisted of 32% capelin, 60% herring, 4% mackerel and 4% salmon. The mean and SE were bootstrapped 100 times. Layer 1 is closest to muscle, while layer 10 is closest to skin.

| **Full blubber - Full blubber CCs - Dietary FA (21) - KL distance** | | | | | | | |
| --- | --- | --- | --- | --- | --- | --- | --- |
|  | Killer whale - SW080429 | Killer whale - SW100500 | Killer whale - SW100743 | Killer whale - SW100830 | Mean ± SE | % Error | Total % Error |
| Capelin | 26.23 | 40.01 | 45.18 | 21.56 | 33.24 ± 6.52 | 3.89 | 21.72 |
| Herring | 71.59 | 59.99 | 43.48 | 72.72 | 61.95 ± 7.15 | 3.24 |  |
| Mackerel | 2.18 | 0.00 | 0.00 | 5.72 | 1.97 ± 2.43 | 50.65 |  |
| Salmon | 0.00 | 0.00 | 11.34 | 0.00 | 2.84 ± 3.07 | 29.09 |  |
| **Layer 1 blubber - Layer 1 blubber CCs - Dietary FA (21) - KL distance** | | | | | | | |
|  | Killer whale - SW080429 | Killer whale - SW100500 | Killer whale - SW100743 | Killer whale - SW100830 | Mean ± SE | % Error | Total % Error |
| Capelin | 38.46 | 42.17 | 28.24 | 44.91 | 38.45 ± 5.63 | 20.14 | 18.11 |
| Herring | 61.54 | 54.31 | 56.67 | 48.82 | 55.33 ± 5.34 | 7.78 |  |
| Mackerel | 0.00 | 3.52 | 12.08 | 0.00 | 3.90 ± 3.01 | 2.48 |  |
| Salmon | 0.00 | 0.00 | 3.01 | 6.27 | 2.32 ± 2.80 | 42.02 |  |
| **Layer 1 blubber - Inner blubber CCs - Dietary FA (21) - KL distance** | | | | | | | |
|  | Killer whale - SW080429 | Killer whale - SW100500 | Killer whale - SW100743 | Killer whale - SW100830 | Mean ± SE | % Error | Total % Error |
| Capelin | 52.93 | 58.90 | 44.51 | 60.93 | 54.32 ± 5.34 | 69.74 | 50.67 |
| Herring | 47.07 | 37.84 | 41.48 | 28.15 | 38.63 ± 6.43 | 35.61 |  |
| Mackerel | 0.00 | 0.00 | 6.31 | 0.00 | 1.58 ± 1.80 | 60.54 |  |
| Salmon | 0.00 | 3.26 | 7.70 | 10.93 | 5.47 ± 3.73 | 36.80 |  |
| **Inner blubber - Inner blubber CCs - Dietary FA (21) - KL distance** | | | | | | | |
|  | Killer whale - SW080429 | Killer whale - SW100500 | Killer whale - SW100743 | Killer whale - SW100830 | Mean ± SE | % Error | Total % Error |
| Capelin | 28.68 | 40.05 | 34.19 | 40.52 | 35.86 ± 5.41 | 12.06 | 16.78 |
| Herring | 66.47 | 58.92 | 50.09 | 57.42 | 58.23 ± 5.51 | 2.96 |  |
| Mackerel | 4.85 | 1.03 | 6.94 | 0.00 | 3.21 ± 2.97 | 19.86 |  |
| Salmon | 0.00 | 0.00 | 8.78 | 2.06 | 2.71 ± 3.31 | 32.26 |  |
| **Inner blubber - layer 1 CCs - Dietary FA (21) - KL distance** | | | | | | | |
|  | Killer whale - SW080429 | Killer whale - SW100500 | Killer whale - SW100743 | Killer whale - SW100830 | Mean ± SE | % Error | Total % Error |
| Capelin | 15.71 | 25.11 | 19.03 | 26.63 | 21.62 ± 5.36 | 32.44 | 48.40 |
| Herring | 75.95 | 68.93 | 64.59 | 73.37 | 70.71 ± 5.12 | 17.85 |  |
| Mackerel | 8.34 | 5.96 | 12.50 | 0.00 | 6.70 ± 4.35 | 67.53 |  |
| Salmon | 0.00 | 0.00 | 3.88 | 0.00 | 0.97 ± 2.53 | 75.77 |  |
| **Outer layer blubber - Full CCs - Dietary FA (21) - KL distance** | | | | | | | |
|  | Killer whale - SW080429 | Killer whale - SW100500 | Killer whale - SW100743 | Killer whale - SW100830 | Mean ± SE | % Error | Total % Error |
| Capelin | 8.60 | 0.00 | 0.66 | 0.00 | 2.32 ± 3.43 | 92.76 | 86.00 |
| Herring | 85.86 | 94.78 | 93.13 | 84.66 | 89.61 ± 5.40 | 49.35 |  |
| Mackerel | 5.54 | 5.22 | 6.21 | 15.34 | 8.08 ± 4.56 | 101.88 |  |
| Salmon | 0.00 | 0.00 | 0.00 | 0.00 | <0.01 ± 0.87 | 100.00 |  |
| **Outer layer blubber - Outer layer CCs - Dietary FA (21) - KL distance** | | | | | | | |
|  | Killer whale - SW080429 | Killer whale - SW100500 | Killer whale - SW100743 | Killer whale - SW100830 | Mean ± SE | % Error | Total % Error |
| Capelin | 52.91 | 36.20 | 39.45 | 25.56 | 38.53 ± 6.78 | 20.40 | 25.73 |
| Herring | 47.09 | 63.80 | 53.31 | 61.96 | 56.54 ± 6.58 | 5.77 |  |
| Mackerel | 0.00 | 0.00 | 0.00 | 12.48 | 3.12 ± 3.22 | 22.02 |  |
| Salmon | 0.00 | 0.00 | 7.25 | 0.00 | 1.81 ± 2.59 | 54.72 |  |
| **Outer layer blubber - layer 10 CCs - Dietary FA (21) - KL distance** | | | | | | | |
|  | Killer whale - SW080429 | Killer whale - SW100500 | Killer whale - SW100743 | Killer whale - SW100830 | Mean ± SE | % Error | Total % Error |
| Capelin | 46.65 | 30.89 | 33.42 | 22.61 | 33.39 ± 6.71 | 4.35 | 67.79 |
| Herring | 46.45 | 61.69 | 54.68 | 55.03 | 54.46 ± 6.51 | 9.23 |  |
| Mackerel | 6.90 | 7.42 | 8.21 | 22.36 | 11.22 ± 4.74 | 180.61 |  |
| Salmon | 0.00 | 0.00 | 3.68 | 0.00 | 0.92 ± 2.19 | 76.97 |  |
| **Layer 10 blubber - layer 10 CCs - Dietary FA (21) - KL distance** | | | | | | | |
|  | Killer whale - SW080429-10 | Killer whale - SW100500-10 | Killer whale - SW100743-10 | Killer whale - SW100830-10 | Mean ± SE | % Error | Total % Error |
| Capelin | 51.45 | 26.58 | 41.82 | 0.89 | 30.18 ± 9.98 | 5.68 | 20.16 |
| Herring | 47.02 | 73.42 | 45.06 | 92.55 | 64.52 ± 10.48 | 7.53 |  |
| Mackerel | 1.53 | 0.00 | 1.41 | 6.56 | 2.37 ± 2.74 | 40.67 |  |
| Salmon | 0.00 | 0.00 | 11.72 | 0.00 | 2.93 ± 3.23 | 26.77 |  |
| **Layer 10 blubber - Outer layer CCs - Dietary FA (21) - KL distance** | | | | | | | |
|  | Killer whale - SW080429-10 | Killer whale - SW100500-10 | Killer whale - SW100743-10 | Killer whale - SW100830-10 | Mean ± SE | % Error | Total % Error |
| Capelin | 58.34 | 32.33 | 48.02 | 3.14 | 35.46 ± 10.45 | 10.81 | 33.53 |
| Herring | 41.66 | 67.67 | 39.34 | 96.86 | 61.38 ± 11.78 | 2.30 |  |
| Mackerel | 0.00 | 0.00 | 0.00 | 0.00 | <0.01 ± 0.50 | 100.00 |  |
| Salmon | 0.00 | 0.00 | 12.64 | 0.00 | 3.16 ± 3.07 | 21.00 |  |


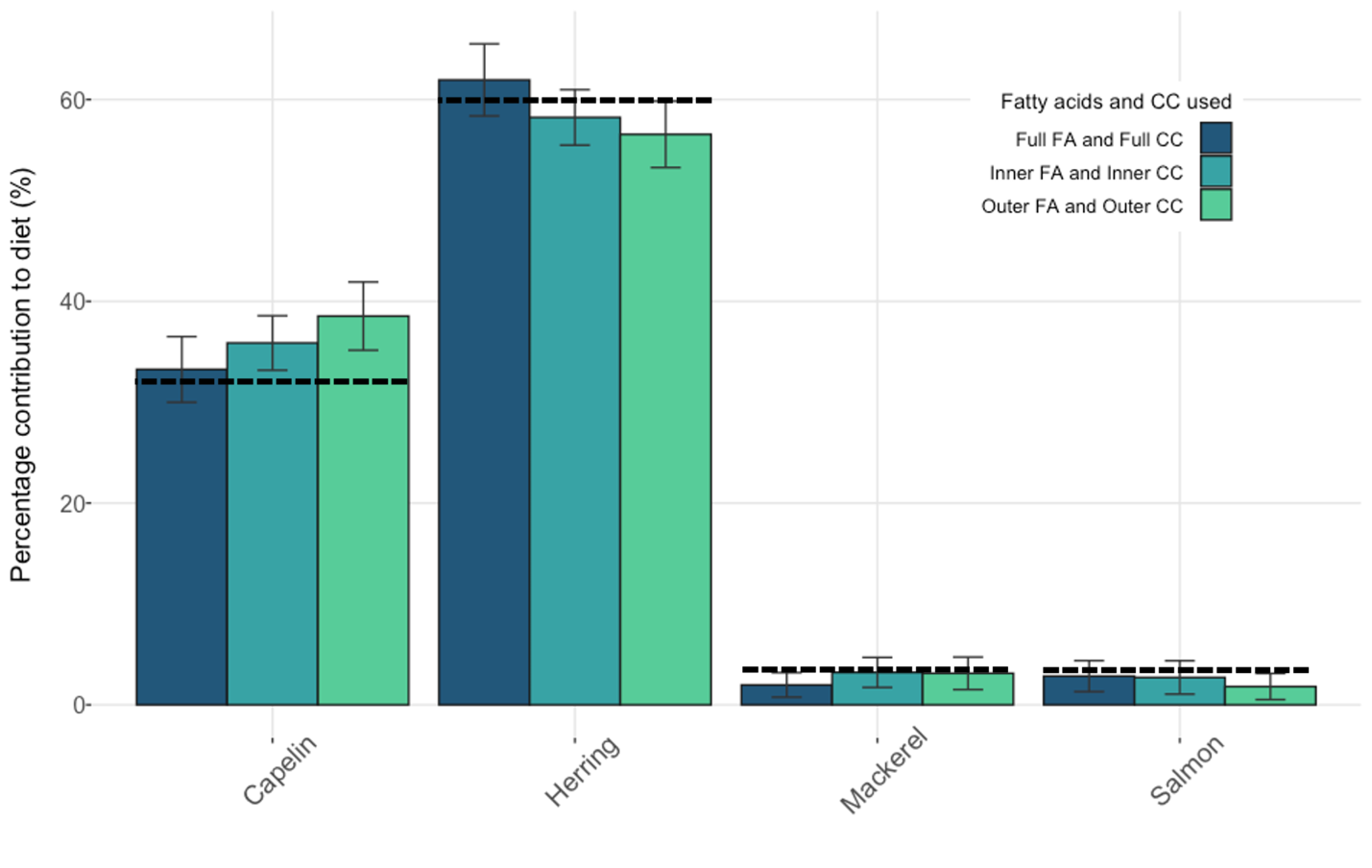
Figure S3: Mean diet estimates (in %) for the four managed-care killer whales when using the average CCs for the four individuals, based on the prey library consisting of capelin (n = 10), herring (n = 10), mackerel (n = 10) and salmon (n = 4). The KL distance was used with the dietary FA set. The true diet (dash line) fed to the managed-care killer whales consisted of 32% capelin, 60% herring, 4% mackerel and 4% salmon.

QFASA validation on the free-ranging killer whales:

Very early on, we tried to include FA signatures from humpback whales (*Megaptera novaeangliae*) as they were identified as potential prey for East Greenlandic killer whales (Rosing-Asvid, pers. Comm.). However, the FA signature from the humpback whale samples we had in our lab varied so much that the humpback whale ellipse overlapped with all other prey species in the PCA, and the species could not be identified during the LOPO analysis. It was thus dropped from the prey library. We found that CCs were essential to calculate QFASA estimates for the wild whales. Additionally, we ran a principal component analysis on the Greenlandic prey library and the Greenlandic killer whales before and after applying the killer whale-derived CCs; and found that applying CCs to the free-ranging whales’ FAs brought the killer whales closer to their potential prey FA ranges (Fig S2). When we ran the *pred_beyond_prey* function on the full depth FA signatures and full blubber CCs, we found that 27% of the killer whales’ FAs were outside the range of the potential prey FAs, while 45% were outside the range of the potential prey FAs without CCs, thus reinforcing the need for CCs in the QFASA modeling approach. We nonetheless ran QFASA on the free-ranging killer whales without CCs as a check and found that the main prey estimated was narwhal (94.28% of the diet), which seemed quite unrealistic, based on the limited stomach content data we obtained for these killer whales. We also selected the dietary FA set over the extended dietary FA set after running the *pred_beyond_prey* function. We found that only 27% of the predator FAs were outside the range of the prey FAs using the dietary FA set while 35% of the predator FAs were outside the range of the prey FAs with the extended dietary FA set, indicating a poorer fit. Additionally, the LOPO estimates were not improved when using the extended FA set. We ran the LOPO with both distances on the dietary FA set and found that the KL distance performed better at separating the prey (Table S5), especially for the “harp and hooded seal” group. Indeed, the KL distance’s percentage of correct attribution to the harp and hooded seal groups was 58% while it was only 37% with the Aitchison distance. Thus, we used the KL distance for diet modeling on the free-ranging killer whales.


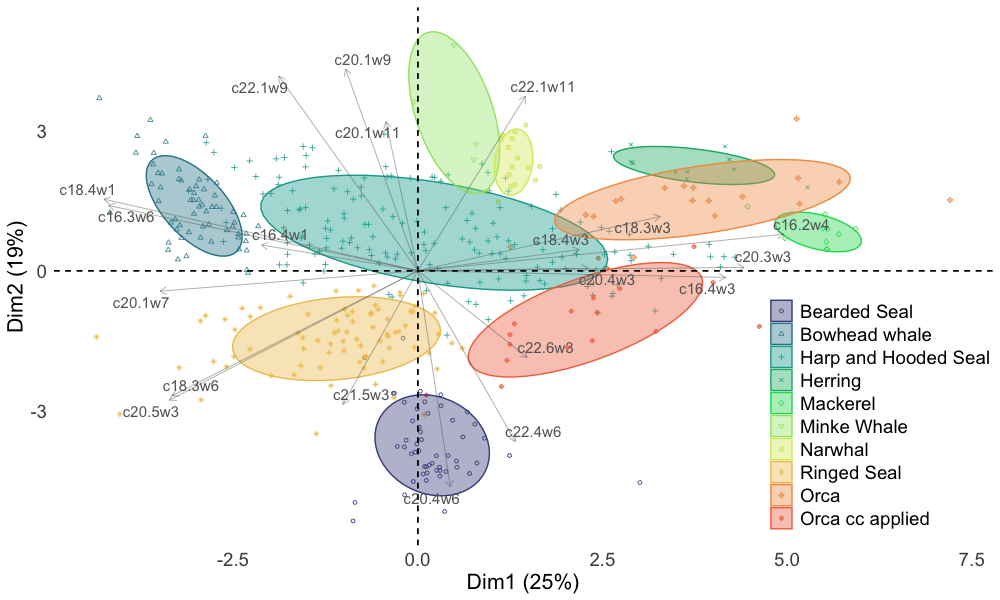


Figure S4: Principal component analysis of dietary FA signatures in prey and Greenlandic killer whales (full blubber) without CCs (in orange) and with CCs (full blubber) generated for this study (in red). Applying CCs to the predator puts them closer to the range of prey FA.

Table S10: Leave-one-prey-out (LOPO) analysis of the Greenlandic prey library using the Aitchison distance and the KL distance on the dietary FA set.

| **Dietary FA - Aitchison distance** | | | | | | | | |
| --- | --- | --- | --- | --- | --- | --- | --- | --- |
|  | Bearded Seal | Bowhead whale | Harp and Hooded Seal | Herring | Mackerel | Minke Whale | Narwhal | Ringed Seal |
| Bearded Seal | **92.15** | 1.30 | 0.80 | 0.02 | 0.25 | 0.45 | 1.55 | 3.47 |
| Bowhead whale | 0.98 | **96.15** | <0.01 | 0.17 | 0.08 | 0.34 | 0.64 | 1.64 |
| Harp and Hooded Seal | 3.75 | 15.50 | **37.08** | 1.62 | 9.40 | 8.08 | 7.39 | 17.19 |
| Herring | 0.24 | 0.43 | <0.01 | **75.91** | 17.63 | 1.40 | 2.94 | 1.46 |
| Mackerel | 0.34 | 0.05 | <0.01 | 6.31 | **92.58** | 0.04 | 0.02 | 0.66 |
| Minke Whale | 1.45 | 1.20 | 1.45 | 0.19 | 3.40 | **72.99** | 11.28 | 8.04 |
| Narwhal | 0.21 | 5.24 | <0.01 | 0.23 | 2.32 | 0.67 | **91.20** | 0.13 |
| Ringed Seal | 5.72 | 3.51 | 0.80 | 0.12 | 0.31 | 0.28 | 1.81 | **87.44** |
|  |  |  |  |  |  |  |  |  |
| **Dietary FA - KL distance** | | | | | | | | |
|  | Bearded Seal | Bowhead whale | Harp and Hooded Seal | Herring | Mackerel | Minke Whale | Narwhal | Ringed Seal |
| Bearded Seal | **90.41** | 0.99 | 1.39 | <0.01 | 0.64 | 0.10 | 3.01 | 3.46 |
| Bowhead whale | 6.96 | **78.71** | 0.45 | 0.49 | 0.32 | 5.94 | 4.48 | 2.65 |
| Harp and Hooded Seal | 3.85 | 6.03 | **58.26** | 0.83 | 5.99 | 8.51 | 3.61 | 12.92 |
| Herring | <0.01 | 0.40 | 0.31 | **81.84** | 11.01 | 2.93 | 1.76 | 1.74 |
| Mackerel | 0.38 | <0.01 | <0.01 | 3.63 | **94.28** | 0.19 | 0.23 | 1.30 |
| Minke Whale | 2.45 | 0.44 | 6.56 | 7.20 | 2.05 | **76.76** | 3.47 | 1.07 |
| Narwhal | 1.65 | 0.42 | 1.94 | <0.01 | 0.15 | 1.66 | **92.98** | 1.20 |
| Ringed Seal | 8.70 | 1.19 | 2.46 | 0.40 | 0.70 | <0.01 | 2.48 | **84.06** |

Table S11: Intra-population variation in QFASA estimates for East Greenland killer whales and relevance of estimates regarding existing stomach content data.

| ID | Geography | Stomach Contents | Season | Date | Sex and Age | Herring | Mackerel | Bearded Seal | Harp and Hooded Seal | Ringed Seal | Narwhal | Minke Whale | Bowhead whale | Total Fish | Total Seal | Total Toothed whale | Total Baleen whale |
| --- | --- | --- | --- | --- | --- | --- | --- | --- | --- | --- | --- | --- | --- | --- | --- | --- | --- |
| 35143 | Greenland | Harp Seal, Minke whale | Summer | 2013 | Adult Female | 15.25 | 13.18 | 30.37 | 21.33 | 0.00 | 19.87 | 0.00 | 0.00 | 28.43 | 51.70 | 19.87 | 0.00 |
| 38340 | Greenland | Harp Seal | Summer | 2012 | Sub-adult | 0.00 | 0.00 | 87.37 | 0.00 | 12.63 | 0.00 | 0.00 | 0.00 | 0.00 | 100.00 | 0.00 | 0.00 |
| 48335 | Greenland | Harp, Hooded seal | Summer | 2012 | Adult Female | 0.00 | 16.39 | 35.98 | 47.63 | 0.00 | 0.00 | 0.00 | 0.00 | 16.39 | 83.61 | 0.00 | 0.00 |
| 48336 | Greenland | Harp Seal | Summer | 2012 | Adult Female | 0.00 | 12.19 | 40.41 | 29.69 | 17.71 | 0.00 | 0.00 | 0.00 | 12.19 | 87.81 | 0.00 | 0.00 |
| 48337 | Greenland | NA | Summer | 2012 | Sub-adult | 0.00 | 16.26 | 48.68 | 25.73 | 9.34 | 0.00 | 0.00 | 0.00 | 16.26 | 83.74 | 0.00 | 0.00 |
| 48338 | Greenland | Harp Seal | Summer | 2012 | Adult Female | 0.00 | 17.95 | 32.81 | 49.24 | 0.00 | 0.00 | 0.00 | 0.00 | 17.95 | 82.05 | 0.00 | 0.00 |
| 48339 | Greenland | NA | Summer | 2012 | Sub-adult | 0.00 | 7.91 | 34.97 | 0.00 | 57.12 | 0.00 | 0.00 | 0.00 | 7.91 | 92.09 | 0.00 | 0.00 |
| 48732 | Greenland | NA | Summer | 2013 | Adult Male | 0.00 | 21.50 | 24.12 | 50.58 | 3.80 | 0.00 | 0.00 | 0.00 | 21.50 | 78.50 | 0.00 | 0.00 |
| 48733 | Greenland | Harp Seal | Summer | 2013 | Adult Female | 0.00 | 9.76 | 49.41 | 0.00 | 40.84 | 0.00 | 0.00 | 0.00 | 9.76 | 90.24 | 0.00 | 0.00 |
| 48735 | Greenland | Harp Seal | Summer | 2013 | Sub-adult | 0.00 | 19.83 | 28.54 | 51.63 | 0.00 | 0.00 | 0.00 | 0.00 | 19.83 | 80.17 | 0.00 | 0.00 |
| 48736 | Greenland | NA | Summer | 2013 | Adult Female | 0.00 | 20.32 | 37.26 | 20.05 | 22.37 | 0.00 | 0.00 | 0.00 | 20.32 | 79.68 | 0.00 | 0.00 |
| 51601 | Greenland | NA | Summer | 2014 | Sub-adult | 0.00 | 23.03 | 27.29 | 15.66 | 34.03 | 0.00 | 0.00 | 0.00 | 23.03 | 76.97 | 0.00 | 0.00 |
| 51606 | Greenland | NA | Summer | 2014 | Sub-adult | 0.00 | 7.14 | 53.41 | 0.00 | 39.45 | 0.00 | 0.00 | 0.00 | 7.14 | 92.86 | 0.00 | 0.00 |
| 51607 | Greenland | NA | Summer | 2014 | Sub-adult | 0.00 | 13.46 | 31.73 | 0.00 | 54.81 | 0.00 | 0.00 | 0.00 | 13.46 | 86.54 | 0.00 | 0.00 |
| 51610 | Greenland | NA | Summer | 2014 | Sub-adult | 0.00 | 7.66 | 41.84 | 0.00 | 40.91 | 9.59 | 0.00 | 0.00 | 7.66 | 82.75 | 9.59 | 0.00 |
| 51613 | Greenland | NA | Summer | 2014 | Sub-adult | 0.00 | 23.83 | 24.32 | 38.84 | 13.01 | 0.00 | 0.00 | 0.00 | 23.83 | 76.17 | 0.00 | 0.00 |
| Bootstrapped (n=100) mean % prey in Greenlandic killer whales' diet (±SE) | | | | | | 1.02 ± 1.10 | 14.51 ± 2.04 | 39.02 ± 5.71 | 22.15 ± 5.44 | 21.38 ± 6.65 | 1.91 ± 1.44 | <0.01 ± <0.01 | <0.01 ± <0.01 | 15.53 ± 1.57 | 82.56 ± 5.93 | 1.91 ± 1.44 | <0.01 ± <0.01 |
| 40888 | Faroe Islands | NA | Winter | 2008 | Adult Female | 0.00 | 27.92 | 30.28 | 0.00 | 41.80 | 0.00 | 0.00 | 0.00 | 27.92 | 72.08 | 0.00 | 0.00 |
| 40889 | Faroe Islands | NA | Winter | 2008 | Sub-adult | 72.57 | 7.76 | 7.98 | 0.00 | 0.00 | 11.69 | 0.00 | 0.00 | 80.33 | 7.98 | 11.69 | 0.00 |
| Bootstrapped (n=100) % prey in Faroese killer whales' diet (±SE) | | | | | | 42.89 ± 24.91 | 16.01 ± 8.21 | 17.10 ± 7.54 | <0.01 ± <0.01 | 17.10 ± 14.15 | 6.91 ± 3.98 | <0.01 ± <0.01 | <0.01 ± <0.01 | 58.89 ± 16.56 | 34.20 ± 7.23 | 6.91 ± 3.98 | <0.01 ± <0.01 |

Bibliography

1 Bourque, J. *et al.* Feeding habits of a new Arctic predator: insight from full-depth blubber fatty acid signatures of Greenland, Faroe Islands, Denmark, and managed-care killer whales Orcinus orca. *Marine Ecology Progress Series* **603**, 1-12, doi:10.3354/meps12723 (2018).

2 McKinney, M. A. *et al.* Global change effects on the long-term feeding ecology and contaminant exposures of East Greenland polar bears. *Global Change Biology* **19**, 2360-2372, doi:10.1111/gcb.12241 (2013).

3 Thiemann, G. W., Derocher, A. E. & Stirling, I. Polar bear Ursus maritimus conservation in Canada: An ecological basis for identifying designatable units. *Oryx* **42**, 504-515, doi:10.1017/S0030605308001877 (2008).

4 Thiemann, G. W., Iverson, S. J. & Stirling, I. Variation in blubber fatty acid composition among marine mammals in the Canadian Arctic. *Marine Mammal Science* **24**, 91-111, doi:<https://doi.org/10.1111/j.1748-7692.2007.00165.x> (2008).

5 Budge, S. M., Springer, A. M., Iverson, S. J., Sheffield, G. & Rosa, C. Blubber fatty acid composition of bowhead whales, Balaena mysticetus: Implications for diet assessment and ecosystem monitoring. *Journal of Experimental Marine Biology and Ecology* **359**, 40-46, doi:<https://doi.org/10.1016/j.jembe.2008.02.014> (2008).

6 Budge, S. M., Iverson, S. J. & Koopman, H. N. Studying trophic ecology in marine ecosystems using fatty acids: A primer on analysis and interpretation. *Marine Mammal Science* **22**, 759-801, doi:10.1111/j.1748-7692.2006.00079.x (2006).

7 Folch, J., Lees, M. & Sloane Stanley, G. H. A simple method for the isolation and purification of total lipides from animal tissues. *J Biol Chem* **226**, 497-509 (1957).

8 Kucklick, J. R. *et al.* Marine mammal blubber reference and control materials for use in the determination of halogenated organic compounds and fatty acids. *Analytical and Bioanalytical Chemistry* **397**, 423-432, doi:10.1007/s00216-010-3596-9 (2010).
